# Bearing Single-Source Domain Generalization Fault Diagnosis Method Based on Adaptive Frequency-Domain Augmentation and Unsupervised Contrastive Learning

**DOI:** 10.3390/s26175349

**Published:** 2026-08-24

**Authors:** Kaisheng Deng, Ping Qu

**Affiliations:** 1 School of Electrical and Automation Engineering, East China Jiaotong University, Nanchang 330013, China; 2 School of Mechatronics and Vehicle Engineering, East China Jiaotong University, Nanchang 330013, China; 2023031001000208@ecjtu.edu.cn

**Keywords:** bearing fault diagnosis, single-source domain generalization, frequency-domain differentiated augmentation, unsupervised contrastive learning, variable operating conditions

## Abstract

Cross-domain distribution shifts severely degrade the diagnostic performance of rolling bearing models under unseen variable operating scenarios. Single-source domain generalization (SDG) builds fault diagnosis models using only single-source vibration data, which fits the practical limitations of industrial data collection. Existing contrastive learning methods adopt uniform spectral perturbations for data augmentation, which easily corrupt fault harmonic characteristics and require massive, labeled training samples. To tackle these drawbacks, this paper proposes an unsupervised contrastive learning framework named FDACL. An adaptive frequency-domain augmentation (AFA) module equipped with learnable weights is designed to separate fault-critical frequency bands from noise components. Differentiated amplitude perturbations are applied to two categories of spectral signals to generate diverse pseudo-samples while retaining intrinsic fault information. A shared encoder is trained with combined InfoNCE contrast loss and classification loss to learn domain-invariant fault representations. Validations are carried out on three datasets, namely Case Western Reserve University (CWRU), Paderborn University (PU), and the industrial CRRC Qingdao Sifang railway wheelset bearing dataset acquired from physical test benches. FDACL achieves average cross-speed diagnostic accuracies of 92.68% and 77.85% on CWRU and PU, respectively, and maintains competitive performance on the Qingdao Sifang industrial dataset. It outperforms state-of-the-art baselines by 4.23–8.71% across all SDG transfer tasks. Ablation experiments and hyperparameter analysis verify the efficacy of the AFA module and contrastive learning scheme, providing an unsupervised diagnostic approach for railway bearings under unknown working conditions.

## 1. Introduction

Intelligent operation and maintenance technologies have been increasingly adopted for critical mechanical equipment such as high-speed trains and aircraft [1]. Rolling bearings serve as core components in these mechanical systems and are typically subjected to harsh operating conditions. Their health status directly governs the reliability and safety of the entire piece of equipment. Therefore, accurate monitoring and timely diagnosis of bearing faults are essential for ensuring efficient and safe industrial production [2].

From the perspective of technical evolution, existing bearing fault diagnosis technologies can be classified into three typical categories. The first category refers to pure signal processing algorithms that exclude machine learning frameworks, which adopt wavelet transform, variational mode decomposition, and other time-frequency analysis tools to extract manual fault features with favorable interpretability; yet, they lack self-adaptability under variable working loads and rotating speeds [3]. The second type is simulation-driven machine learning diagnosis, which establishes multi-body dynamic and finite element models to generate abundant labeled vibration samples for compensating the scarcity of measured fault data, while inherent distribution discrepancies exist between simulated signals and real test rig measurements [4]. The third branch corresponds to physics-model-based intelligent diagnosis, which embeds bearing kinematic laws and vibration dynamic equations into neural networks to improve model interpretability, yet most existing implementations are built upon supervised training and require multi-domain labeled datasets [5]. Each of the three technical routes has its own applicable scenarios and unavoidable drawbacks, which have promoted continuous innovation of intelligent diagnostic algorithms.

In recent years, breakthroughs in deep learning have advanced the application of artificial intelligence in the automatic fault detection and identification of mechanical equipment [6]. Deep-learning-based bearing fault diagnosis methods have achieved nearly 100% classification accuracy on multiple public datasets. Nevertheless, these approaches rely on a critical assumption that the training and test data follow identical data distributions. In practical industrial scenarios, variations in ambient conditions, rotating speeds, and loads inevitably lead to distribution shifts between datasets, which drastically degrade the performance of pre-trained models on unseen working conditions and severely limit their practical engineering applicability [7].

Domain adaptation represents the mainstream solution to mitigate distribution shifts, yet it requires target-domain samples during the training phase [8]. Given the diversity of operating conditions for industrial machinery, it is impractical to collect vibration signals covering all potential working scenarios in advance, which creates a data acquisition bottleneck for domain adaptation methods. By contrast, domain generalization (DG) algorithms require no target-domain data for training and aim to construct models capable of generalizing directly to unseen domains, making them more suitable for real engineering deployments [9]. Existing DG research predominantly focuses on multi-source DG, which trains models by leveraging data collected from multiple distinct source domains [10]. However, collecting multi-source datasets incurs high costs and poses implementation obstacles in most industrial sites. Accordingly, single-source domain generalization (SDG) techniques that only demand data from one source domain possess greater practical potential [11]. The core distinction between single-source and multi-source DG lies in the quantity of training source domains [12]. Although SDG imposes stricter constraints and brings greater challenges to algorithm design, it imposes looser data requirements and aligns better with real industrial demands [13].

At present, SDG has been extensively investigated in computer vision and natural language processing, while relevant studies in the field of fault diagnosis predominantly focus on multi-source DG. For instance, Wang et al. [14] proposed DGNetMSAC, which decouples domain-related features via domain-specific auxiliary classifiers and convolutional autoencoders, and improves generalization performance by adopting a domain-invariant classifier. Shen [15] introduced a semantic-discriminative augmentation method to address imbalanced DG. Zhao et al. further established a standardized benchmark for cross-domain fault diagnosis to systematically evaluate the performance of various existing algorithms [16].

In contrast, research on SDG for machinery fault diagnosis remain scarce. Zhao and Shen put forward AMINet, the pioneering SDG model in fault diagnosis, which addresses distribution shifts via a domain generation module and mutual information minimax game [17]. Kim et al. [18] constructed the SDGPI framework that integrates signal processing priors to enhance model generalization and interpretability. Wang et al. developed MSGACN to generate diverse feature representations relying on style transfer and adversarial learning [19]. Nevertheless, all the above methods are developed under supervised learning paradigms and require abundant labeled samples. To date, unsupervised SDG approaches for fault diagnosis have rarely been explored, leaving an obvious research gap in this field [20].

Based on the above literature review, the main research gaps and limitations of existing single-domain generalization (SDG) fault diagnosis approaches can be summarized as follows:

(1) Most time series contrastive learning frameworks adopt universal random augmentation strategies for vibration signals. These generic augmentation operations treat all frequency components equally, which may destroy fault-related spectral harmonic features and lose critical fault information when generating augmented samples.
(2) Most existing single-domain generalization diagnostic methods belong to supervised learning paradigms, which require abundant accurately annotated fault samples for model training. In practical industrial scenarios, collecting large-scale labeled vibration data is labor-intensive and costly, which severely restricts the real-world applicability of such supervised solutions.
(3) Current single-domain generalization fault diagnosis approaches rarely differentiate fault-critical spectral components from noise-dominated interference components in the frequency domain. An identical perturbation intensity is imposed over the full frequency spectrum, which weakens the feature extraction capacity and degrades generalization performance under unknown working conditions.

To address the aforementioned research gaps, this paper presents an unsupervised contrastive learning framework named FDACL. Specifically, we design an adaptive frequency-domain augmentation module equipped with a learnable mechanism that automatically distinguishes fault-sensitive spectral components from irrelevant frequency bands. Differentiated amplitude modulation is implemented on the two categories of components to simulate diverse shifts in operating conditions, which yields sufficient pseudo-target samples exclusively using single-source domain data. Moreover, the proposed augmentation strategy is embedded within an unsupervised contrastive learning pipeline. The model minimizes the feature distance between each raw sample and its augmented view while maximizing the feature discrepancy across dissimilar samples, enabling it to extract domain-invariant feature embeddings robust to variable working conditions and realize single-source domain generalization (SDG) for rolling bearing fault diagnosis. Thanks to the massive pseudo-target samples produced by the augmentation module, the model can accurately approximate latent target data distributions even when only limited source training data are available, and it retains a strong generalization capability under small-sample circumstances. The main contributions of this work, addressing the above-mentioned research gaps correspondingly, are summarized as follows:

(1) To address the heavy annotation dependency of supervised single-domain generalization (SDG) fault diagnosis methods mentioned in Gap 1, an unsupervised contrastive-learning framework named Frequency-Domain Adaptive Contrastive Learning (FDACL) has been constructed. The proposed framework learns discriminative fault representations without relying on large-scale labeled vibration samples, which improves the practicability for real industrial bearing diagnosis scenarios.
(2) To address the drawback of indiscriminate universal augmentation in existing contrastive-learning pipelines described in Gap 2, an adaptive frequency-domain augmentation (AFA) module has been designed. It generates augmented vibration samples while preserving fault-related spectral harmonic features, avoiding the loss of critical fault information during data augmentation.
(3) In response to the limitation that existing approaches apply uniform perturbation over the full frequency spectrum, as illustrated in Gap 3, the AFA module implements differentiated amplitude perturbation for fault-critical spectral components and noise-dominated interference components. This mechanism enhances the capability to extract domain-invariant fault features and boosts diagnostic generalization performance under unseen working conditions.

The remainder of this paper is organized as follows. Section 2 introduces the basic theories of single-source domain generalization and unsupervised contrastive learning. Section 3 elaborates on the proposed FDACL framework and its core adaptive frequency-domain augmentation module. Section 4 presents comprehensive experimental verification, including experimental configuration, baseline algorithms, ablation studies, and three case studies on the CWRU, Paderborn University (PU), and CRRC Qingdao Sifang wheelset bearing datasets. Section 6 conducts a horizontal performance comparison between the proposed FDACL and other state-of-the-art bearing diagnosis methods reported in the recent literature. Section 7 summarizes the overall findings, discusses the inherent limitations of our method, and prospects future research directions.

## 2. Related Theory

This chapter introduces the basic theories closely related to this work, including single-source domain generalization and unsupervised contrastive learning. The definitions and fundamental principles are provided in the following subsections, which lay the theoretical basis for the proposed FDACL method.

### 2.1. Multi-Source and Single-Source Domain Generalization

**Figure 1:**
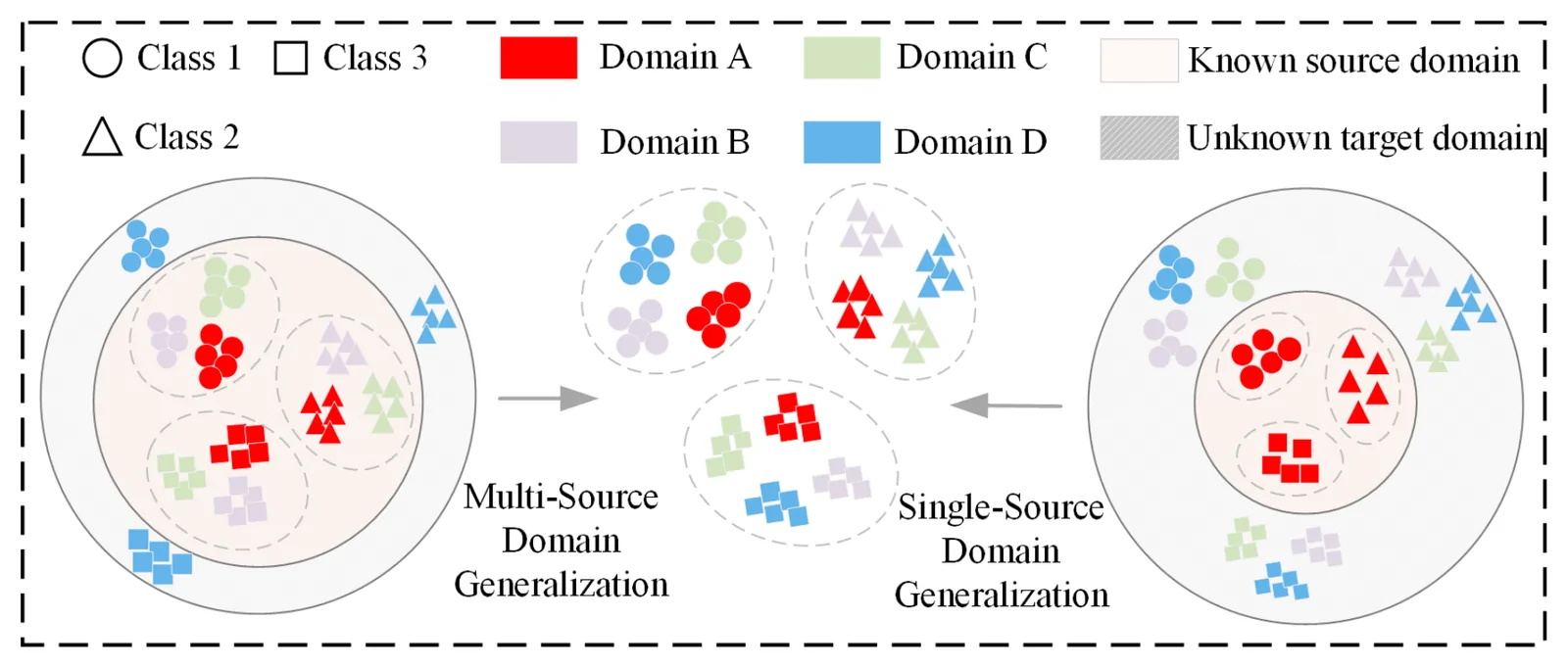
Conceptual schematic of multi-source domain generalization and single-source domain generalization tasks.

DG aims to build models capable of generalizing to unseen target domains [21]. Since target-domain data are unavailable during training, extracting domain-invariant features from limited source-domain information constitutes its core challenge. Current mainstream research mostly centers on the multi-source domain generalization (MDG) paradigm, which integrates heterogeneous data from multiple source domains and exploits complementary cross-domain information to boost model generalization. Nevertheless, constrained by practical factors such as data acquisition costs and privacy regulations, multi-source datasets are difficult to acquire steadily in real-world scenarios, which hinders the practical deployment of relevant methods. In contrast, SDG completes model training using merely one source domain, presenting superior advantages in data collection and practical deployment and better conforming to real application demands. As illustrated in Figure 1, the essential distinction between MDG and SDG lies in the composition of training data: the former learns from multiple heterogeneous source domains, whereas the latter relies solely on a single source domain. Correspondingly, SDG is subject to stricter constraints when mining transferable shared features across domains, resulting in substantially higher generalization difficulty than MDG. Despite greater technical obstacles, SDG has emerged as a vital research branch of DG oriented toward practical applications due to its lower data dependence and higher practical value.

### 2.2. Unsupervised Learning

**Figure 2:**
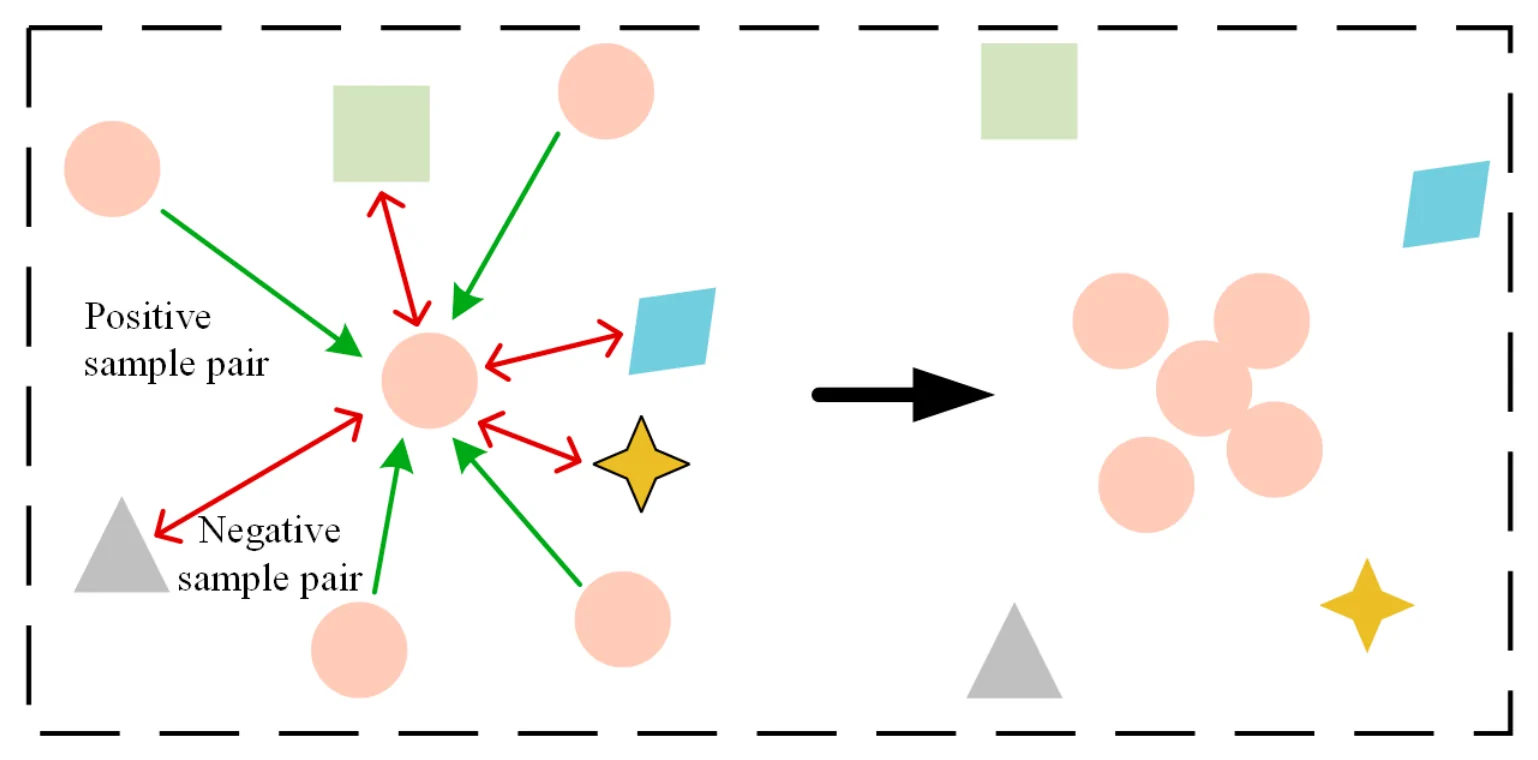
Conceptual schematic diagram of unsupervised contrastive learning.

Unsupervised learning is a training paradigm that eliminates manual labeling requirements, enabling low-cost utilization of massive unlabeled datasets. Unlike limited supervision signals provided by sparse labeled samples, unlabeled data fully retains intricate patterns and inherent correlations within raw data distributions, laying a solid foundation for learning universal data representations. Contrastive learning stands as one of the core frameworks for unsupervised representation learning and has achieved remarkable progress in fault diagnosis, time series analysis, and other research areas [22]. Its core mechanism lies in training feature encoders by optimizing contrastive loss functions. Specifically, the loss function constrains the encoder to minimize the feature distance between positive sample pairs (semantically identical instances) while maximizing the distance between negative sample pairs (semantically distinct instances). By clustering similar samples and separating dissimilar ones in the feature space, contrastive learning can learn discriminative and transferable feature representations. A schematic illustration of contrastive learning is presented in Figure 2.

Considering the challenges of expensive data annotation for industrial vibration time series, contrastive learning has achieved great success in general time series representation learning tasks [23]. A series of representative self-supervised contrastive frameworks has been proposed for generic time series modeling. TS2Vec [24] performs hierarchical contrastive learning to capture timestamp-wise robust representations for arbitrary-length time series. Temporal Neighborhood Coding (TNC) [25] builds temporal contrastive objectives to distinguish temporally neighboring signals from irrelevant non-neighboring segments. TS-TCC [23] designs a cross-view prediction auxiliary task to enhance the consistency of feature embeddings between two augmented views of raw time series. MHCCL [26] further introduces masked hierarchical clustering to refine temporal contrastive representation learning for multivariate sequential data. Although these pioneering works adopt diverse network architectures and training objectives, the core principle of contrastive learning remains consistent: pulling positive pairs (views from the same original signal) close and pushing negative pairs (samples from different fault modes/working conditions) apart in latent feature space [9]. Accordingly, high-quality view generation via data augmentation becomes an indispensable prerequisite for guaranteeing effective contrastive training.

For mechanical vibration signal analysis, InfoNCE loss is naturally compatible with the above contrastive learning paradigm and particularly suitable for bearing cross-condition fault diagnosis. On one hand, InfoNCE implements instance-wise contrast without extra category-level supervision, which matches the unsupervised single-source training setting of this paper where all source-domain samples are unlabeled. On the other hand, the cosine similarity metric embedded in InfoNCE can precisely quantify the feature discrepancy between original vibration spectra and augmented pseudo-condition samples generated by our adaptive frequency-domain augmentation module. The positive pairs constructed by spectrum-differentiated perturbation retain consistent fault characteristic semantics, while negative pairs naturally carry domain shift differences caused by rotating speeds and loads. Optimizing InfoNCE loss can therefore drive the encoder to filter out domain-specific interference and excavate intrinsic fault-discriminative features invariant to varying working conditions.

Within the framework of unsupervised contrastive learning, the construction of positive and negative pairs heavily relies on data augmentation strategies [27]. Positive samples are generally generated by applying mild augmentation operations to raw signals, whereas negative samples are produced via aggressive augmentation transformations or directly sampled from other instances in the training set. For one-dimensional vibration signals, mainstream augmentation approaches include random Gaussian noise injection, random amplitude scaling, random time stretching and random cropping. Owing to its unique advantages, unsupervised contrastive learning has exhibited prominent application prospects in time series research fields plagued by high labeling costs.

## 3. Proposed Method

On the basis of the above theory, this section describes the architecture of the proposed FDACL in detail. The adaptive frequency-domain augmentation module and the overall contrastive learning pipeline are elaborated in separate subsections.

### 3.1. Adaptive Frequency-Domain Augmentation Module

**Figure 3:**
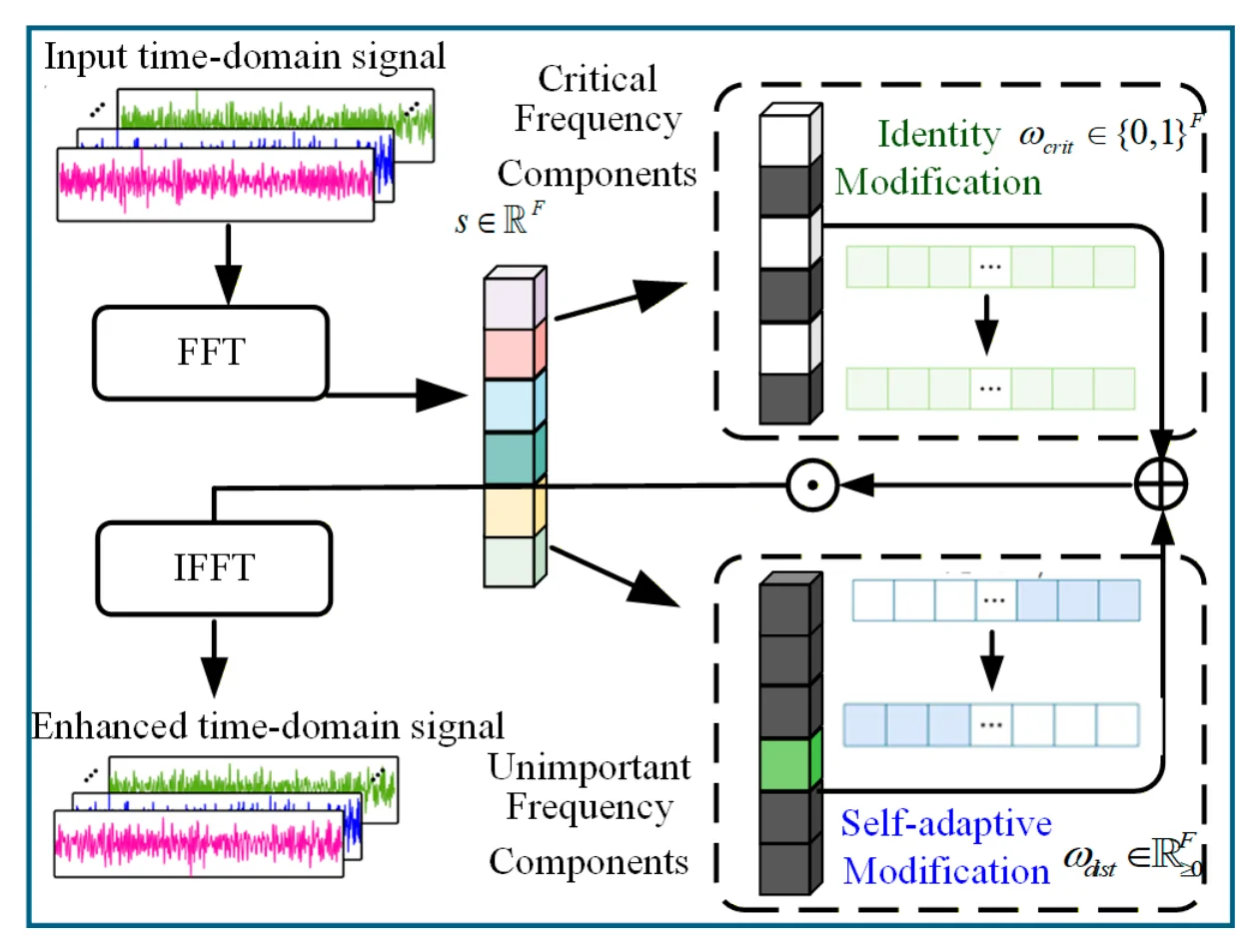
Adaptive frequency-domain augmentation module.

To fully exploit the global characteristics of mechanical vibration signals in the frequency domain and address the drawbacks of conventional fixed augmentation strategies, including severe corruption of fault semantics and insufficient discriminability of features learned via unsupervised learning, this paper proposes an adaptive frequency-domain augmentation (AFA) module, the schematic diagram of which is illustrated in Figure 3.

Let the raw one-dimensional vibration signal with length L be defined as x(t). The discrete Fourier transform (DFT) is first applied to convert the time-domain signal into its spectral form:

(1) $$x_{f}=F(x)\in {\mathbb{C}}^{F}$$

where F(x) denotes the discrete Fourier transform, and $F=\left\lfloor L/2\right\rfloor +1$ represents the number of effective frequency bins.

Leveraging the orthogonality and conjugate symmetry of Fourier basis functions, complete signal features can be retained by merely processing the first F frequency-domain components. To quantify the contribution of each frequency component to fault discrimination, a learnable weight vector for frequency components is introduced:

(2) $$s={\left[s_{1},s_{2},\ldots ,s_{F}\right]}^{T}\in {\mathbb{R}}^{F}$$

The sigmoid function is applied to normalize the weight vector to the interval (0, 1), yielding the importance weight of each frequency bin:

(3) $$w_{f}=\sigma (s_{f})=\frac{1}{1+\exp(-s_{f})},\;f=1,2,\ldots ,F$$

Here, a value of $w_{f}$ close to 1 indicates that the corresponding frequency component contains abundant fault-sensitive information, while a value approaching 0 implies that the component is dominated by noise and redundant information. A threshold τ is adopted to partition frequency components into a core component set $F_{c}$ and a non-core component set $F_{n}$:

(4) $$\left\{\begin{array}{l} F_{c}=\{f_{i}|\sigma (s_{i})>\tau \}\\ F_{n}=\{f_{i}|\sigma (s_{i})\leq \tau \} \end{array}\right.$$

In the developed AFA module, τ is set as a fixed predefined hyperparameter rather than a learnable variable updated via backpropagation during model training, with a default value of 0.5 adopted throughout all experiments in this work. This static threshold design eliminates the extra computational overhead caused by optimizing an additional segmentation parameter and maintains a stable spectrum partition criterion throughout the entire training procedure. During iterative optimization, only the lightweight weight vector s is updated continuously to adjust the normalized importance score $\sigma (s_{i})$ of each spectral bin, while τ acts merely as a fixed discriminant boundary to separate fault-sensitive characteristic frequencies and trivial noise-dominated frequency components.

The end-to-end optimization objective jointly optimizes two categories of trainable parameters: the weight matrices of the feature extraction encoder backbone and the lightweight frequency importance weight vector s for spectral component evaluation. All parameters are updated synchronously via a unified Adam optimizer without introducing gradient constraints for the segmentation threshold τ.

A dynamic augmentation mask M is generated according to component importance, and correction vectors $\omega_{crit}$ for core frequency components and $\omega_{dist}$ for non-core frequency components are constructed separately to implement differentiated augmentation on the two types of components:

(5) $$M_{f}=\left\{\begin{array}{lll} U(0.9,1.1), & f\in F_{c} & \left(w_{\mathrm{crit}}\right)\\ U(1/hp,hp), & f\in F_{n} & \left(w_{\mathrm{dist}}\right) \end{array}\right.$$

where U(⋅) stands for uniform distribution over a bounded real interval. For fault-critical spectral components, the amplitude scaling factor is sampled uniformly within the narrow range [0.9,1.1], introducing mild fluctuations to retain intrinsic fault feature information without distortion. For non-critical spectral components dominated by operating-condition interference and random noise, a wider uniform interval is adopted to simulate drastic spectral discrepancies across unseen working domains. In all experimental tasks of this work, the hyperparameter hp is fixed to 2, so the random scaling coefficients for non-core components follow U(0.5,2). The broad variation range strengthens the diversity of generated pseudo cross-domain samples and improves the model’s robustness against unknown load and speed variations. $w_{\mathrm{crit}}$ imposes slight amplitude perturbations on core fault components to prevent the destruction of critical features, whereas $w_{\mathrm{dist}}$ introduces large-amplitude disturbances to non-core components to enhance the model’s robustness against varying working conditions and noise.

Accordingly, the augmented frequency-domain representation can be uniformly formulated as follows:

(6) $${\tilde{x}}_{f}=x_{f}⊙(w_{\mathrm{crit}}+w_{\mathrm{dist}})=x_{f}⊙M$$

In Equation (6), ⊙ denotes element-wise multiplication. The augmented time-domain signal is recovered via an inverse discrete Fourier transform:

(7) $$\tilde{x}=F^{-1}({\tilde{x}}_{f})$$

After the above adaptive frequency-domain augmentation, the proposed module strictly preserves the semantic integrity of critical fault characteristic frequencies while introducing spectral disturbances correlated with variable working loads and rotating speeds. This procedure generates pseudo-target domain samples carrying cross-domain discrepancy characteristics, which further facilitates the construction of semantically consistent yet distribution-diverse contrastive views for subsequent unsupervised contrastive learning. The augmentation module is jointly optimized end-to-end with the feature encoder during training. It requires neither target-domain data nor manually predefined prior knowledge of fault frequency bands. Instead, the module autonomously learns the importance of each frequency component and dynamically adjusts augmentation intensity, providing core support for the model to extract domain-invariant fault feature representations with strong robustness.

### 3.2. Frequency-Domain Differentiated Augmentation Contrastive Learning Module

**Figure 4:**
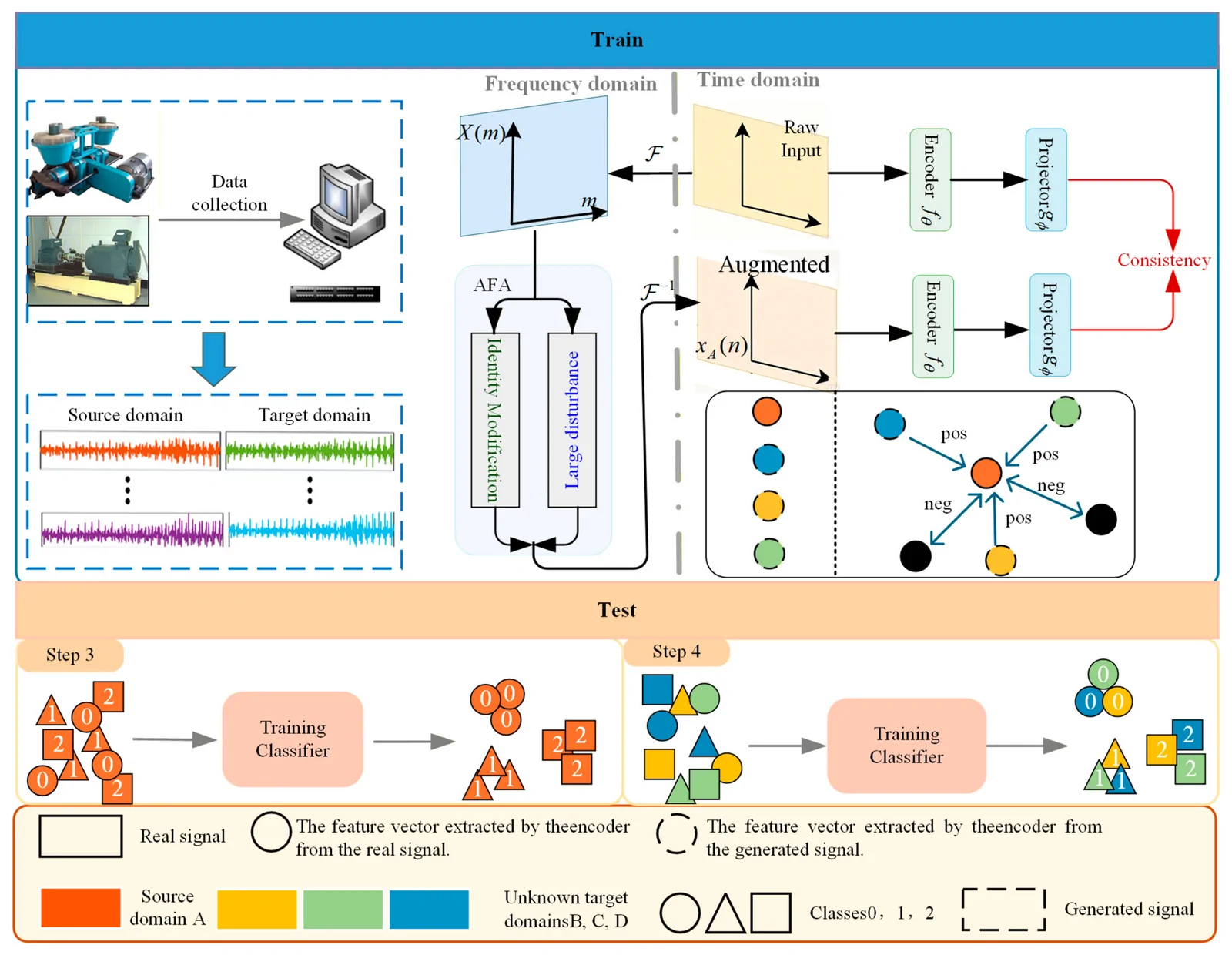
Overall framework of the proposed FDACL.

To address the insufficient generalization performance of bearing fault diagnosis models under single-source domain settings when deployed to unseen working conditions, this paper proposes an unsupervised contrastive learning model termed FDACL. The overall framework of FDACL is depicted in Figure 4, which consists of four key modules: frequency-domain representation construction, adaptive frequency-domain augmentation, feature encoding and contrastive learning, and downstream fault diagnosis. The proposed framework can learn cross-condition robust features by utilizing only unlabeled source-domain data.

First, raw vibration time series signals are fed into the model and mapped to the frequency domain via fast Fourier transform, to explicitly extract fault-related characteristic frequency information of bearings. Based on the obtained frequency-domain representations, an adaptive frequency-domain augmentation module is introduced to impose learning-driven, differentiated perturbations on the spectrum. Relying on a frequency component importance learning mechanism, this module adaptively distinguishes fault-critical and non-critical frequency components. Mild perturbations are applied to critical components to preserve consistent fault semantics, while intensive large-amplitude disturbances are imposed on non-critical components to simulate spectral variations under diverse operating conditions. Consequently, pseudo-target domain samples with inter-domain discrepancies are generated from source-domain raw samples.

Subsequently, the original frequency-domain sample $x_{f}$ and its augmented counterpart ${\tilde{x}}_{f}$ are simultaneously fed into a parameter-shared feature encoder $f_{\theta }(\cdot )$ to generate the corresponding feature representations:

(8) $$z_{0}=f_{\theta }\left(X_{f}\right),\;z_{1}=f_{\theta }\left({\tilde{X}}_{f}\right)$$

The extracted features will then be fed into a two-layer projector to obtain projection vectors for InfoNCE loss calculation during pre-training. In the unsupervised contrastive learning stage, an instance-wise InfoNCE contrastive loss is adopted to regularize the learned feature representations:

(9) $$L_{\mathrm{con}}=-\log\frac{\exp\left(\mathrm{sim}\left(z_{0},z_{1}\right)/T\right)}{\sum_{k=1}^{B}\exp\left(\mathrm{sim}\left(z_{0},z_{k}\right)/T\right)}$$

where sim(⋅,⋅) denotes the cosine similarity function, B represents the batch size, and T is the temperature coefficient. This loss function enforces consistency between different frequency-domain views derived from an identical signal in the feature space, while enlarging the feature distance between distinct samples. It thereby guides the encoder to extract domain-invariant feature representations robust to variations in working conditions.

To avoid an extreme distribution of the frequency scoring vector and to prevent failure in core component identification, a weight regularization term is incorporated into the total loss function:

(10) $$L_{\mathrm{reg}}=\lambda \cdot \frac{1}{F}\sum_{f=1}^{F}w_{f}$$

where λ denotes the balance hyperparameter that controls the contribution of the weight regularization term to the total loss. This hyperparameter is tuned via preliminary experiments on the validation set. Subsequent concise quantitative experiments are carried out to reveal the correlation between this regularization coefficient and cross-domain diagnostic performance. The final overall loss is formulated as follows:

(11) $$L_{\mathrm{total}}=L_{\mathrm{con}}+L_{\mathrm{reg}}$$

After the unsupervised pre-training is finished, all parameters within the feature encoder *f_θ_* are completely frozen and no longer updated. Only a lightweight one-layer linear classifier is introduced for downstream fault-classification tasks. The frozen encoder projects input frequency-domain signals into fixed-dimension domain-invariant features, and cross-entropy loss is utilized to optimize only the weights and biases of the linear classifier. The AdamW optimizer, learning rate, and training epochs for this linear classifier are set identically to those adopted by supervised baselines for fair comparison. During the inference phase, our framework requires neither labeled samples from target domains nor additional fine-tuning on the encoder. Reliable fault diagnosis across varying rotational speeds and loads can be achieved relying merely on the model pre-trained by single-source-domain data.

To clearly summarize the complete training and inference workflow of the proposed FDACL method, we provide the step-by-step algorithm implementation in Algorithm 1, which systematically records all core operations, including data preprocessing, adaptive frequency-domain augmentation, contrastive loss optimization, and a two-stage training strategy.
**Algorithm 1** Pseudocode of FDACL**Input**: Source-domain vibration dataset $D_{s}$, total training epochs E**Output**: Well-trained FDACL model for unseen target-domain fault diagnosis1: Preprocess: Segment raw one-dimensional vibration signals into sample sequences2: Initialize feature encoder, AFA module, classification head with random weights3:   **for** epoch = 1 to E
**do**4:     Sample mini-batch samples from source-domain dataset $D_{s}$
5:     Generate augmented signal views via Adaptive Frequency-Domain Augmentation6:     Extract hierarchical domain-invariant features by dual-stream feature encoder7:     Compute instance contrastive loss $L_{con}$
8:     Compute AFA regularization loss $L_{reg}$
9:     Calculate total loss $L_{total}=L_{con}+L_{reg}$
10:     Update network parameters by minimizing $L_{total}$ with Adam optimizer11:   **end for**12:  Freeze all parameters of the pre-trained feature encode $f_{\theta }$
13:  Train lightweight one-layer linear classifier using cross-entropy loss14: **Inference**: For unseen target-domain samples, extract features and predict fault labels15: **return** Trained FDACL model

Benefiting from the above elaborate design, FDACL effectively models complex variations such as shifting rotational speeds without prior target-domain data or label information. Trained solely on data collected from a single working condition, the proposed method maintains stable and superior fault-classification accuracy when generalized to unseen operating domains.

## 4. Materials

This section fully introduces all experimental supporting materials adopted in this paper, including three bearing vibration datasets, hardware and software experimental platforms, and unified data preprocessing pipelines. Sample waveform visualizations and detailed parameter tables of each dataset are provided in corresponding subsections for clear reproducibility.

### 4.1. Experimental Hardware and Software Platform

All experiments are implemented using the PyTorch 1.13.1 deep learning framework with CUDA 11.8 for GPU acceleration. The hardware platform consists of an Intel 64 Family 6 Model 140 Stepping 1 CPU and an NVIDIA GeForce MX450 graphics card. To eliminate the influence of random fluctuations, each experiment is repeated 10 times, and the average results are recorded for analysis.

### 4.2. Data Preprocessing Pipeline

Before model training, unified preprocessing operations are applied to all raw vibration signals. The raw one-dimensional vibration sequences are segmented into non-overlapping samples with a fixed length of 1024 points. A fast Fourier transform (FFT) is then performed to convert time-domain waveforms into frequency-domain spectra, which are used as the input to the model. No additional filtering or denoising operations are artificially introduced, so as to preserve the original industrial noise features and realize realistic simulation of cross-domain distribution shifts.

### 4.3. Case Western Reserve University Bearing Dataset

**Figure 5:**
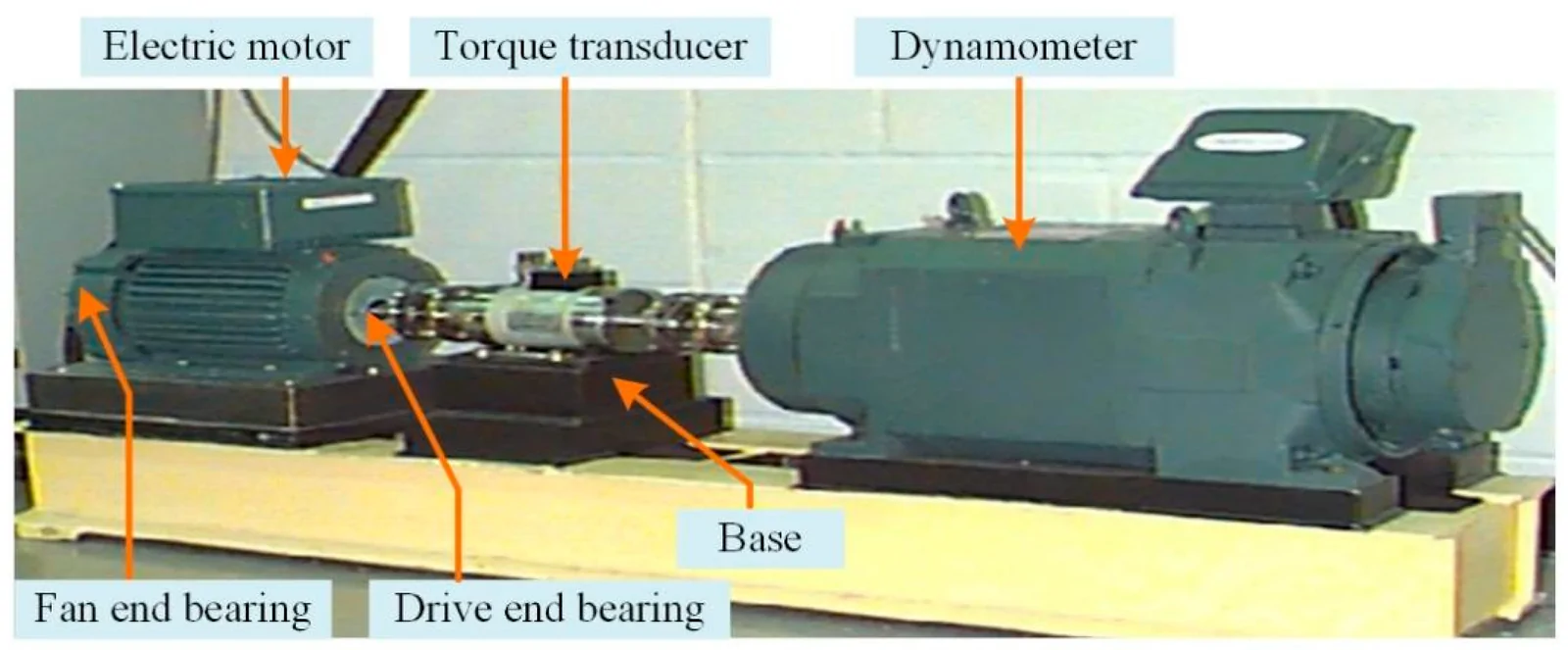
CWRU test rig.

Figure 5 illustrates the structure of the experimental test rig for Case Western Reserve University (CWRU) bearing fault diagnosis. This platform mainly consists of core components, including an electric motor, torque transducer, mounting base, dynamometer, and electronic controller, which are utilized to collect bearing vibration signals.

Experiments are carried out under four distinct rotational speeds to simulate practical variable-speed operating conditions. The dataset covers four types of bearing health states: normal (NA), inner race fault (IR), outer race fault (OR), and rolling element fault (RF). For each fault mode, three different damage severities are configured, with corresponding defect sizes of 0.1778 mm, 0.3556 mm and 0.5334 mm, as detailed in Table 1.

In this work, vibration signals collected from the drive-end bearing with a sampling frequency of 12 kHz are adopted. The dataset contains a total of 10 fault categories, comprehensively covering combinations of various rotational speeds and fault severities. It is well-suited for evaluating the performance of fault diagnosis algorithms under variable-speed working conditions.

### 4.4. Paderborn University Bearing Dataset

**Figure 6:**
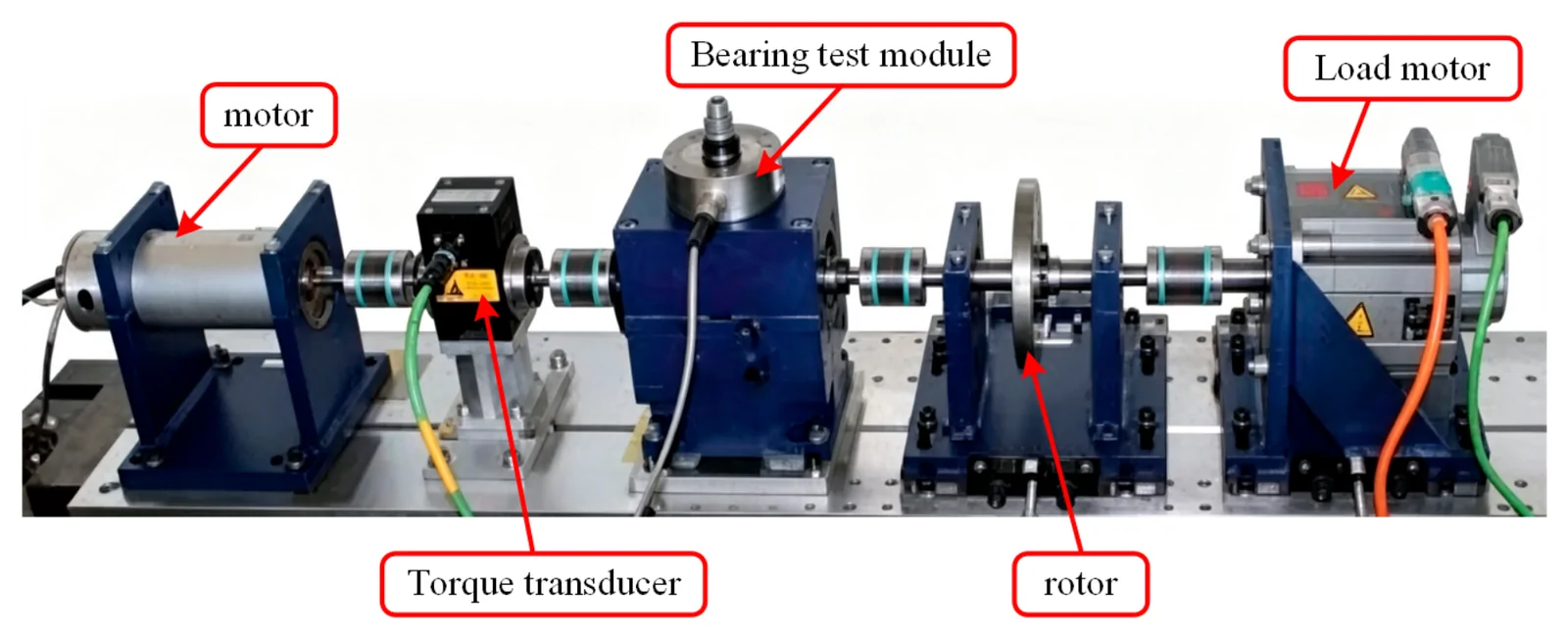
PU dataset test rig.

To further validate the generalization performance of the proposed FDACL method and eliminate the potential reliability concerns raised by the limitations of the CWRU dataset, additional experiments are conducted on the Paderborn University (PU) bearing dataset. The PU dataset contains real bearing fault samples acquired from accelerated lifetime tests conducted on a modular bearing test rig, as illustrated in Figure 6, which provides more realistic and industrially relevant fault scenarios compared to artificially seeded fault samples. The test rig consists of an electric motor, a torque transducer, a bearing test module, a rotor, and a load motor. Vibration signals are collected at a sampling frequency of 64 kHz. Six health conditions of rolling bearings are selected for experimental evaluation, including the normal condition, inner race fault, outer race fault, and rolling element fault with different damage severities.

Four distinct operating conditions are included in the PU dataset, with variations in rotational speed, load torque, and radial force. The detailed configuration of each operating domain is summarized in Table 2. Compared with the CWRU dataset, where speed variations are relatively minor and load changes are applied in a less controlled manner, the PU dataset exhibits more significant domain shifts across different operating conditions, making it a more challenging and reliable benchmark for evaluating domain generalization methods.

### 4.5. Wheelset Bearing Dataset from CRRC Qingdao Sifang

**Figure 7:**
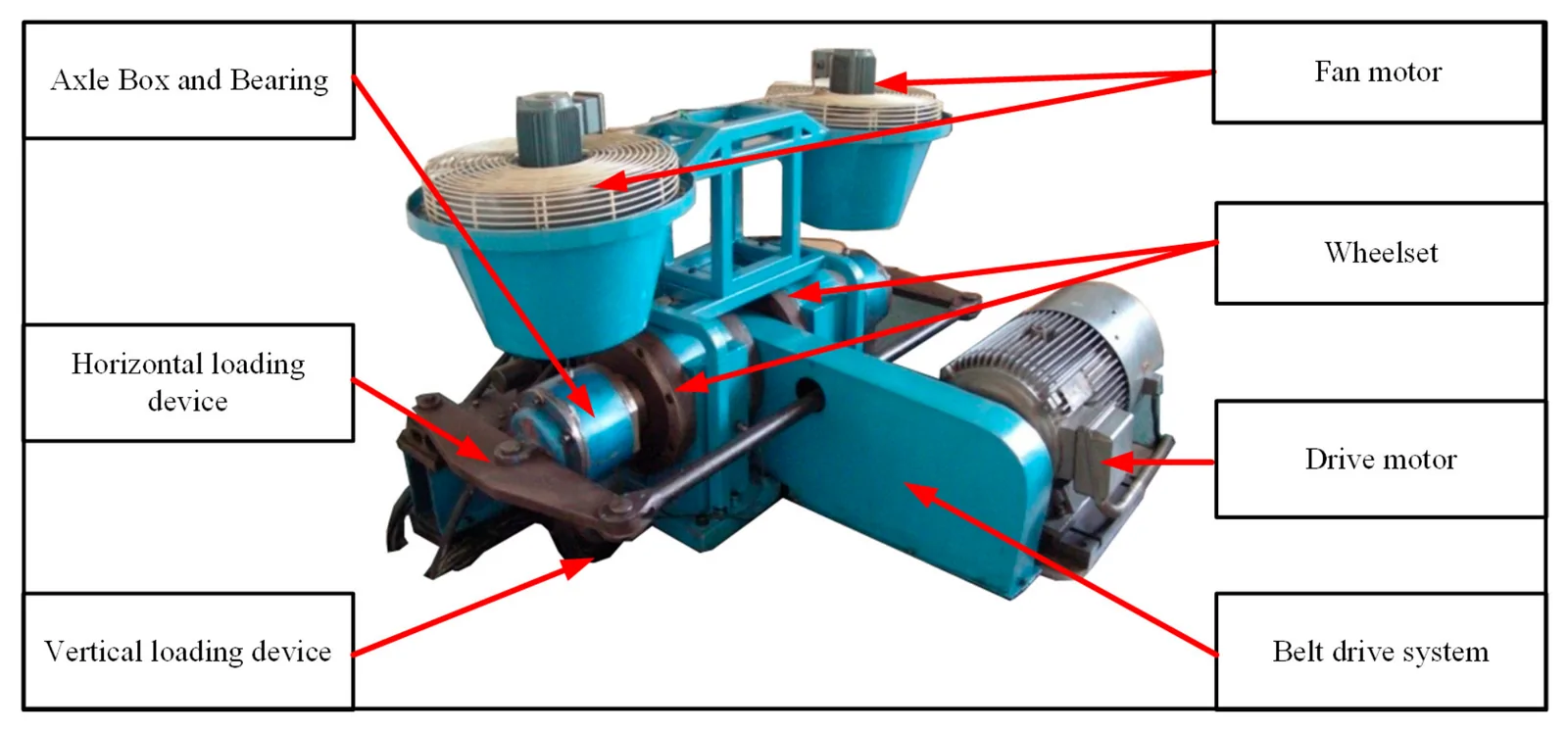
Test rig of Qingdao Sifang wheelset bearing.

The configuration of the CRRC Qingdao Sifang wheelset bearing test rig adopted in this case study is illustrated in Figure 7. The platform mainly consists of key components, including a cooling fan motor, drive motor, wheelset axle, belt transmission system, test bearing, and horizontal and vertical loading devices. By coordinately adjusting these components, diverse operating states of bearings can be simulated, providing a reliable experimental platform for accurate acquisition of wheelset bearing vibration signals.

To reproduce practical variable-speed operating conditions, the bearing is operated under three distinct rotational speeds. Four bearing health states are configured on the test rig, namely normal condition, inner race fault, outer race fault and rolling element fault, so as to comprehensively evaluate the variable-speed fault diagnosis capability of the proposed method.

**Figure 8:**
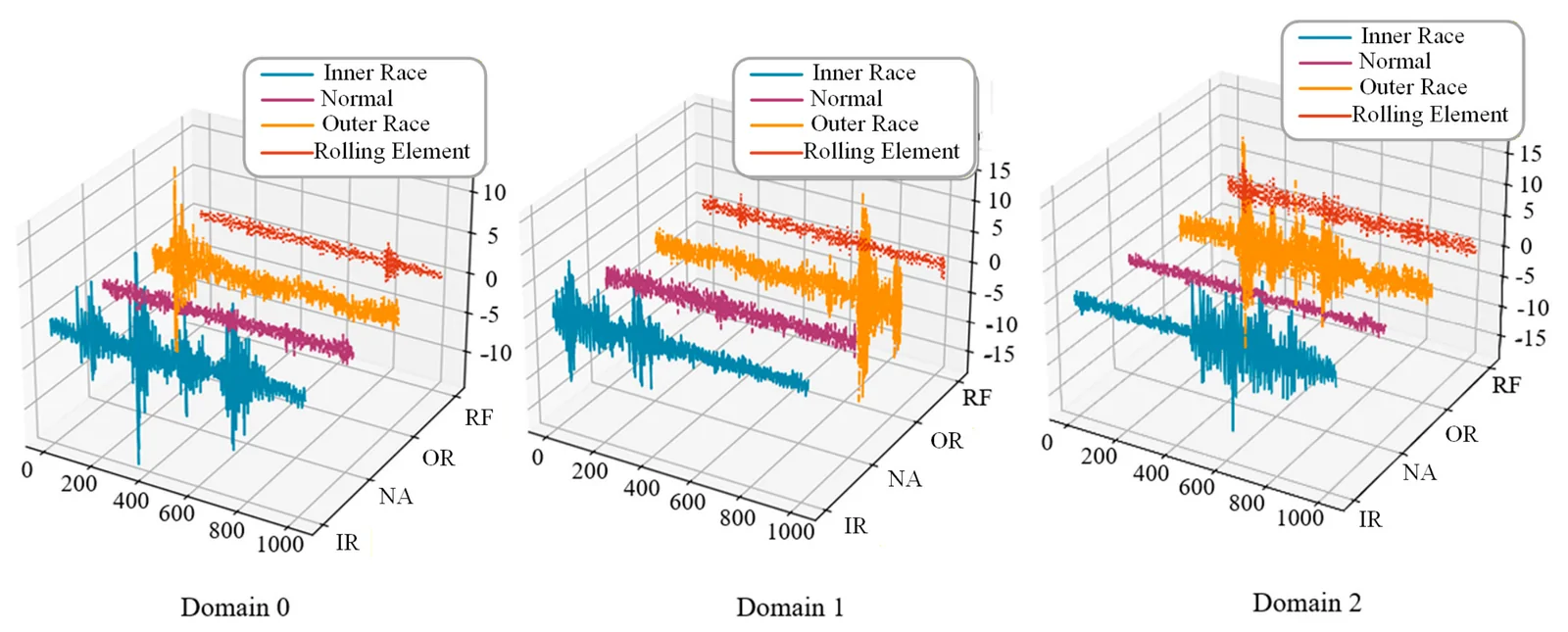
Visualization of vibration signals across different domains.

Detailed parameters of the bearing dataset are summarized in Table 3. Figure 8 displays raw vibration waveforms of wheelset bearings under three distinct working conditions (D0–D2). Obvious discrepancies in amplitude fluctuation characteristics and frequency distributions can be observed among signals collected under different operating conditions and health states, which distinctly reflect the data distribution shifts induced by varying working conditions.

## 5. Experimental Verification and Result Analysis

This chapter conducts comprehensive quantitative and qualitative analyses to verify the generalization, robustness and computational efficiency of the proposed FDACL method. Comparative experiments against state-of-the-art baselines, small-sample tests, feature visualization, computational overhead statistics, hyperparameter sensitivity analysis, and systematic ablation studies are presented in separate subsections.

### 5.1. Baseline Methods and Implementation Details

In this subsection, we compare the proposed FDACL with several classic and state-of-the-art DG approaches. Detailed descriptions of all comparative baselines are presented below:

(1) Empirical risk minimization (ERM): ERM serves as the fundamental baseline and is solely trained with standard cross-entropy loss [28].
(2) Invariant risk minimization (IRM): IRM leverages causal inference tools to formalize the mathematics of spurious and invariant correlations. It mitigates the over-reliance of machine learning systems on data biases, enabling stable generalization to unseen test distributions [29].
(3) Risk extrapolation (REx): Proposed in [30], REx alleviates distribution shifts by minimizing risk discrepancies across training domains to reduce the model’s sensitivity to extreme variations during deployment.
(4) Conditional contrastive domain generalization (CCDG): This method aims to maximize mutual information among samples of identical categories collected from distinct domains, while simultaneously minimizing mutual information between samples belonging to different fault classes [31].
(5) Style-agnostic networks (SagNet): SagNet decouples domain-style encodings from categorical representations to avoid prediction bias induced by domain styles, forcing the model to prioritize intrinsic content features instead of domain-specific styles [32].
(6) Deep Domain Confusion (DDC): A classic unsupervised domain adaptation baseline based on feature distribution alignment. DDC introduces Maximum Mean Discrepancy (MMD) loss to narrow the feature distribution gap between source and target domains without accessing any target label information. This approach matches the unsupervised training paradigm of our FDACL framework. We adopt DDC for a fair cross-paradigm comparison between self-supervised contrastive learning and traditional unsupervised feature alignment strategies [33].
(7) Hilbert Envelope Spectrum + SVM (HSVM): We perform the Hilbert transform on raw vibration signals to extract fault-impulse-sensitive envelope spectra, then manually construct multi-dimensional feature vectors combining time-domain statistics, frequency-domain amplitudes and envelope spectral indicators. A support vector machine (SVM) with a radial basis function (RBF) kernel is utilized as the classifier for fault identification. This baseline is included to compare the adaptive feature learning superiority of FDACL against fixed handcrafted signal features [34].

**Figure 9:**
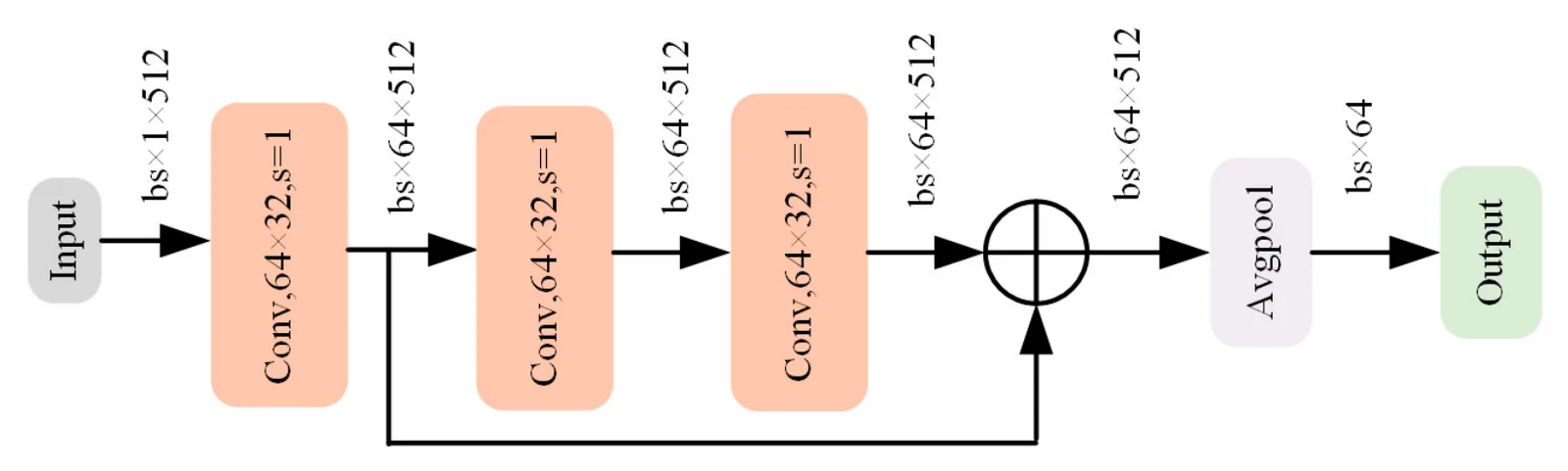
Structure of the simplified ResNet backbone network.

Among the above baselines, ERM, IRM, REx, CCDG, and SagNet fall into supervised deep learning frameworks; DDC is an unsupervised deep domain adaptation method; and Hilbert Envelope Spectrum + SVM represents the conventional signal processing diagnostic route. To guarantee fair, consistent and credible quantitative evaluation, we unify the network architecture, training hyperparameters and dataset preprocessing rules for all deep-learning-based competitors, and align the raw signal input rules for the traditional signal processing pipeline:

(1) FDACL and all deep learning baselines adopt the same simplified ResNet backbone illustrated in Figure 9, followed by a unified single-layer fully connected linear classifier for final fault classification.
(2) Hyperparameter settings: We apply unified training hyperparameters across all models. The batch size is set to 64, the total number of training epochs is 300, the initial learning rate is 0.01, and the AdamW optimizer is consistently utilized.
(3) Training pipeline division:

Supervised DG baselines (ERM, IRM, REx, CCDG, SagNet): The backbone encoder and linear classifier are trained end-to-end for 300 epochs using fully labeled source-domain data.

Unsupervised deep learning methods (DDC, FDACL): No target labels are involved during training. For FDACL, the encoder backbone undergoes 300 epochs of self-supervised pre-training with unlabeled source signals; all backbone weights are frozen after pre-training, and only the single-layer linear classifier is further trained for another 300 epochs under identical optimization settings. The classifier fine-tuning stage involves far less computational overhead than end-to-end training of supervised methods. We fix the training iterations of the classification head to eliminate unfair comparisons caused by inconsistent fine-tuning steps.

By standardizing all experimental constraints above, we eliminate performance discrepancies originating from mismatched network structures, hyperparameters, or input data. The average diagnostic accuracy obtained under such unified settings can objectively reflect the intrinsic cross-domain generalization capacity of each algorithm.

### 5.2. Case Study 1: CWRU Bearing Dataset

#### 5.2.1. Experimental Results and Analysis

To comprehensively evaluate the performance of the proposed FDACL, two experimental test scenarios are designed in this paper.

Scenario 1 (sufficient training samples): In this scenario, 80% of the samples from the source domain are allocated to the training set to simulate a circumstance with abundant training data.

Scenario 2 (limited small samples): To mimic the practical challenge of scarce training data, the number of samples per fault category was gradually reduced to construct a small-sample learning setting.

From the perspective of practical industrial deployment, an important experimental design is adopted: apart from the training samples, all remaining source-domain samples are incorporated into the target-domain test set. This design ensures that the model maintains stable performance on the original source domain while achieving favorable generalization ability toward unseen target domains, which better satisfies the requirements of real-world engineering deployment.

(1) Scenario 1 (sufficient training samples): Under the experimental setup with abundant training samples, a complete cross-load transfer diagnostic task suite is constructed based on the CWRU dataset. The detailed partition of all transfer tasks is summarized in Table 4. All tasks uniformly perform classification for 10 bearing health and fault states (C0–C9). The diagnostic accuracy results of the 12 transfer tasks are presented in Table 5, where the best performance for each task is highlighted in bold, and the second-best result is underlined.

As shown in Table 5, the proposed FDACL does not rank first on every single cross-domain transfer task of the CWRU dataset, yet it delivers prominent and robust generalization performance, especially on the most difficult transfer scenarios. First of all, FDACL attains the optimal diagnostic accuracy on tasks A10, A11, and A12, which are recognized as high-challenge cross-working-condition transfer tasks with obvious distribution shifts between source and target domains. For other tough tasks with severe performance degradation of contrastive methods, such as A2 and A3, FDACL achieves the top-two accuracy among all deep learning baselines and significantly outperforms the traditional signal processing pipeline HSVM (Hilbert Envelope Spectrum + SVM) by a large margin. Notably, ERM, IRM, REx, CCDG, SagNet and DDC are all deep learning baselines trained with labeled source data, while HSVM relies on manually handcrafted signal features with supervised SVM classification. In stark contrast, our FDACL operates under an unsupervised pre-training paradigm and does not utilize any fault label information in the entire training stage. Even without label supervision, FDACL maintains competitive or even superior diagnostic accuracy compared with all supervised counterparts across most cross-load transfer tasks. In particular, the performance gap between FDACL and all baselines becomes wider on hard tasks (A3, A10, A12) where distribution discrepancy is severe. The traditional HSVM method suffers drastic accuracy decline under cross-domain shifts, since fixed handcrafted signal features fail to capture invariant fault characteristics under varying loads. This fully demonstrates that the proposed FDACL can automatically mine robust frequency-domain invariant representations via self-supervised contrastive learning, verifying its strong generalization ability and superiority for bearing fault diagnosis under variable operating conditions.

**Figure 10:**
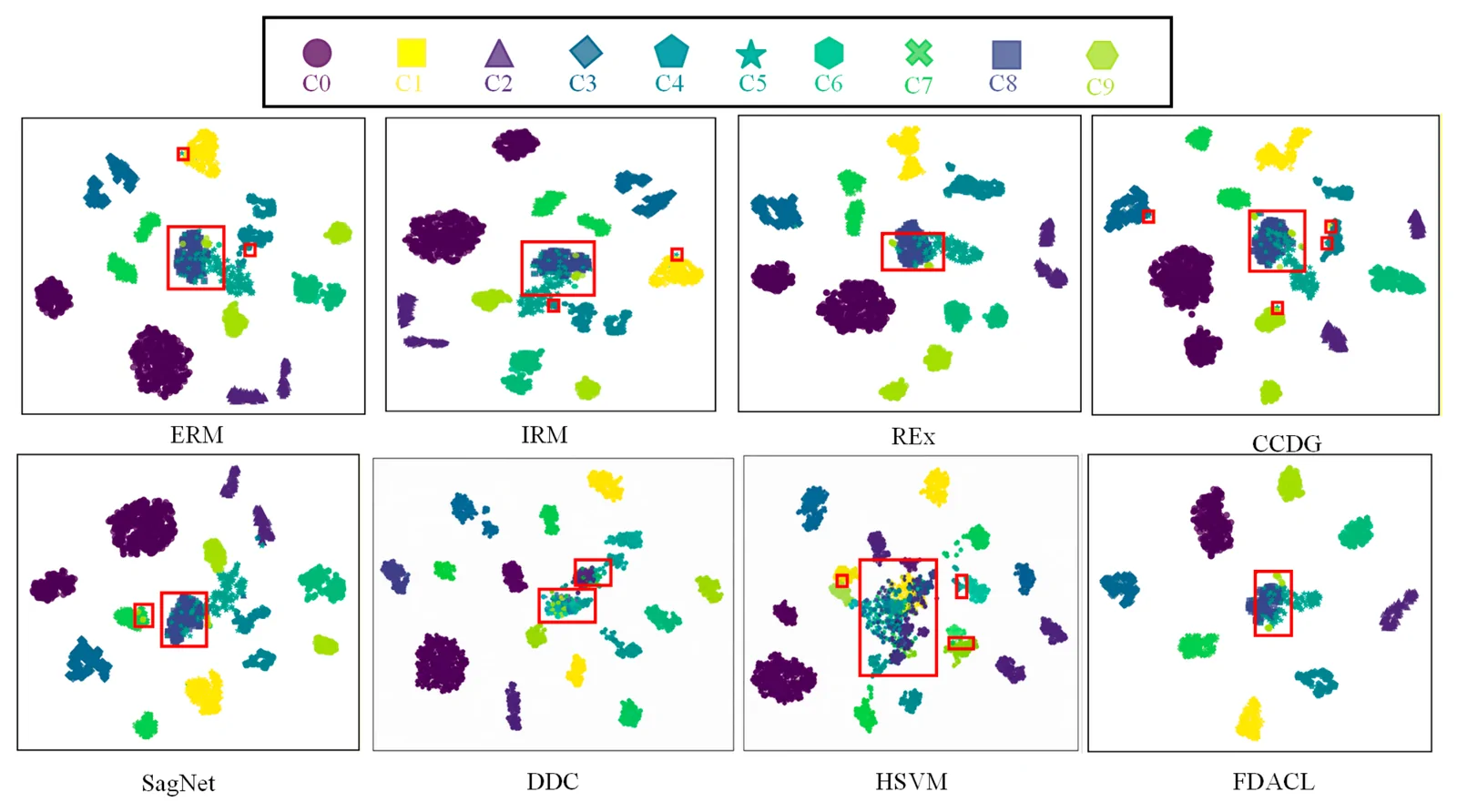
t-SNE visualization results under transfer task A10.

We employ the t-SNE dimensionality reduction technique to visualize the fault feature representations learned by all competing methods. Figure 10 illustrates the feature distribution of the challenging cross-load transfer task A10, which enables an intuitive visual comparison of the feature clustering quality across different algorithms. For the supervised baseline ERM, the extracted features exhibit severe global scattering. Most fault categories form loose clusters, and obvious overlap appears among samples belonging to C0, C1 and C4, making it impossible to generate compact, well-separated feature regions. IRM and REx deliver marginal improvements in intra-class aggregation, but prominent cross-domain dispersion persists. Samples corresponding to minor fault types spread over a wide range, and the boundaries between distinct fault clusters remain blurred and intertwined. CCDG and SagNet obtain a tighter intra-class feature concentration relative to the above methods, yet they lack sufficient discriminative capacity for highly similar fault patterns. Severe sample mixing and cluster misalignment can be observed for categories C2 and C5, and the overall feature clusters still maintain loose structures.

By contrast, the features learned by our proposed FDACL achieve outstanding aggregation performance for identical fault samples collected under disparate load conditions. Each fault category forms compact, complete and independent feature clusters. The samples of C0, C1 and C4 are clearly separated into isolated concentrated regions, which greatly suppresses feature scattering caused by cross-domain distribution shifts and minimizes inter-class overlapping areas.

In particular, C2 and C5 share highly analogous fault signatures, leading to extensive sample mixing and indistinct cluster boundaries for all baseline approaches. However, FDACL produces far more compact intra-class distributions, negligible cross-domain mixing and drastically fewer ambiguous sample points between these two similar fault modes.

In summary, compared with all supervised deep learning baselines and the traditional signal-processing-based HSVM pipeline, FDACL effectively aligns homogeneous fault features extracted under varying loads and rotating speeds. It yields well-defined cluster structures, tighter within-class feature aggregation, and wider inter-class decision margins. This superior unsupervised cross-domain feature clustering performance visually verifies the powerful invariant feature learning capability of FDACL for cross-load bearing fault diagnosis on the CWRU dataset.

Apart from diagnostic accuracy, computational overhead is a vital metric for practical bearing fault diagnosis. As shown in Table 6, FDACL adopts a two-stage training strategy: the entire encoder backbone is frozen after pre-training, and only the lightweight linear classifier is optimized. Unlike all end-to-end deep learning baselines, FDACL’s efficiency gains only appear in the training phase, while inference FLOPs and total model parameters stay consistent due to the shared backbone. All deep learning baselines (ERM, IRM, REx, CCDG, SagNet, DDC) share identical network structures, yet extra loss functions make them consume more GPU memory and training time than ERM. The traditional HSVM method has the shortest training time without neural networks or GPU support, but it suffers terrible cross-domain generalization. Benefiting from only 0.65 K trainable parameters in the fine-tuning stage, FDACL achieves the lowest training cost among all deep models. Compared with ERM, it cuts peak GPU memory by 43.1% and training time by 15.8% with superior accuracy, realizing a favorable balance between generalization and computational efficiency for industrial deployment.

**Figure 11:**
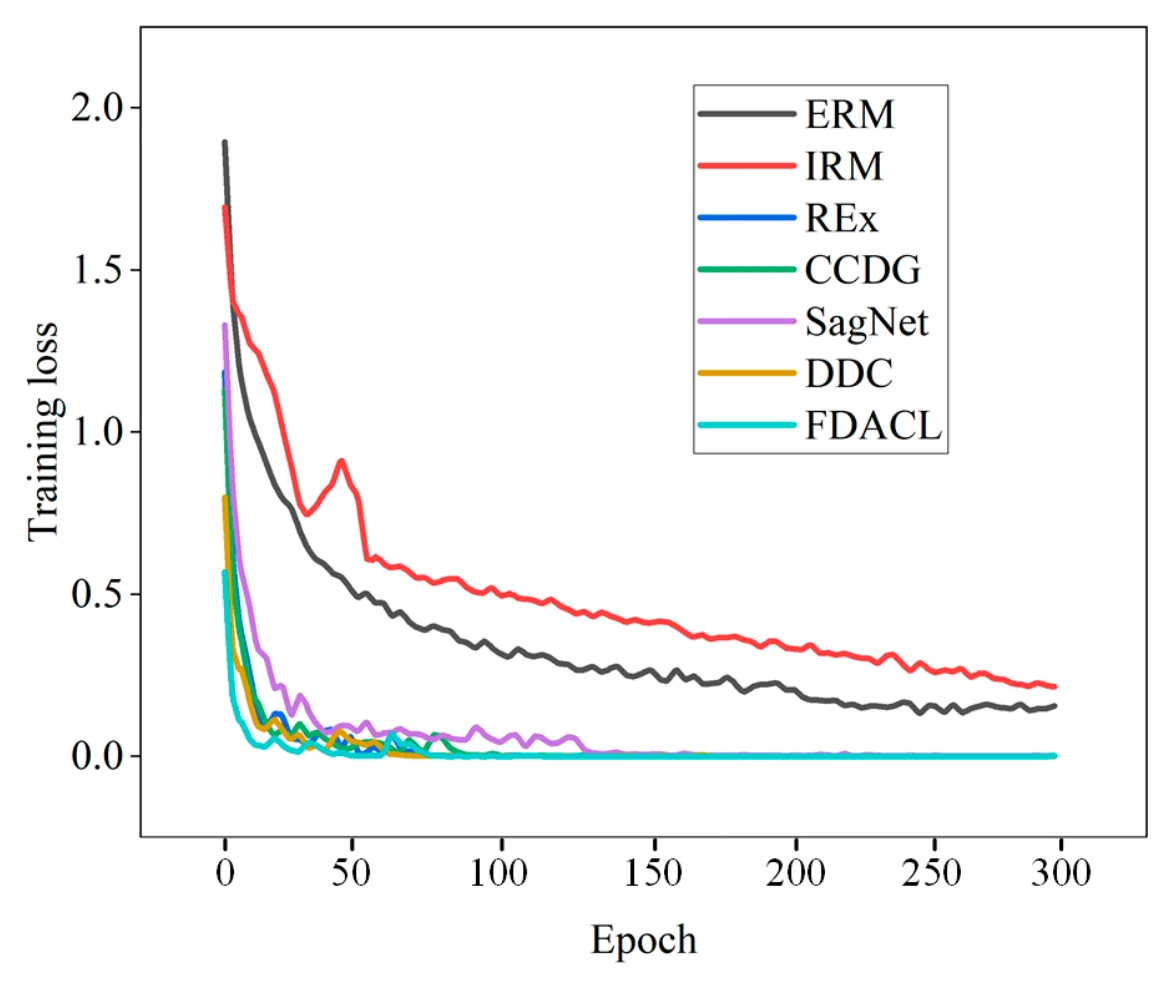
Variation in training loss with training epochs on the CWRU dataset.

We supplement Figure 11 to visualize the training loss convergence characteristics of all deep learning comparison methods on the CWRU dataset. As shown in Figure 11, FDACL achieves faster and more stable loss convergence than all supervised and unsupervised deep baselines, which intuitively verifies the superior optimization efficiency of our two-stage training strategy. Since HSVM is a traditional signal processing-SVM pipeline without iterative epoch training, it cannot be plotted in this loss curve figure, and we provide hyperparameter sensitivity analysis of HSVM as alternative visualization.

The convergence curve in Figure 11 demonstrates that FDACL achieves faster and smoother loss fitting across variable working conditions, enabling rapid model retraining for on-site equipment calibration and reducing maintenance downtime. The confusion matrix error distribution shows that traditional baselines fail to detect incipient bearing micro-faults, which would lead to unplanned shutdowns in practice; FDACL’s superior identification accuracy for subtle damage supports condition-based early maintenance instead of periodic blind disassembly. The narrow 95% confidence interval of FDACL’s test accuracy guarantees stable and reliable monitoring outputs for standardized maintenance scheduling. Overall, the proposed FDACL provides robust, interpretable diagnostic support for rolling bearing predictive maintenance in rail transit equipment, balancing operational safety and maintenance expenditure.

(2) Scenario 2 (small-sample setting): Experiments on the CWRU dataset were carried out to evaluate generalization performance under scarce training data. Domain 3 is set as the source domain, and Domain 1 serves as the target domain.

**Figure 12:**
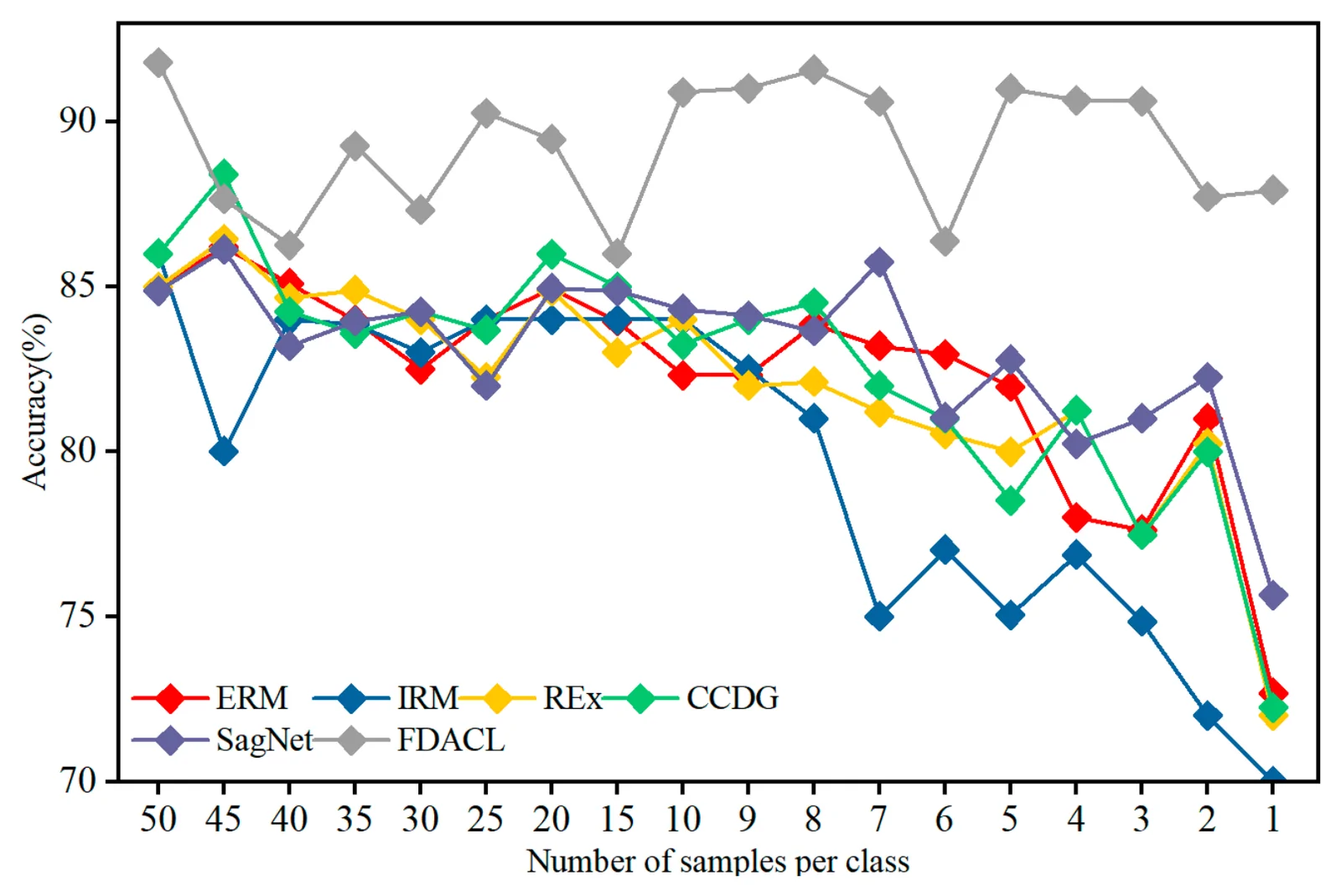
Results for the small sample size case.

To fully simulate practical industrial data-limited scenarios, where labeled bearing fault samples are hard and costly to collect, we gradually reduce the number of training samples drawn from the source domain to construct a series of small-sample test settings. Specifically, we fix the source domain as Domain 3 of the CWRU dataset and extract a limited number of labeled samples for each fault category as training data, where the sample size per class is sequentially set to 50, 45, 40, 35, 30, 25, 20, 15, 10, nine, eight, seven, six, five, four, three, two and, finally, one. The corresponding classification accuracy curves under all sample scales are visualized in Figure 12. As clearly reflected by the plotted curves, the majority of baseline DG models suffer sharp, severe accuracy degradation when the number of labeled samples per fault category shrinks, and the performance drop becomes particularly drastic once the per-class sample quantity falls below 10. In stark comparison, the proposed FDACL framework demonstrates remarkable small-sample generalization robustness with the flattest accuracy curve across the whole sample quantity range. Only minor accuracy loss can be observed even when training with merely one or two samples per fault class. Under all extremely scarce labeled data conditions, FDACL consistently achieves the highest diagnostic accuracy and surpasses all comparative baseline algorithms by a considerable performance gap.

#### 5.2.2. Hyperparameter Sensitivity Analysis

To quantitatively reveal the influence of regularization weight λ on the cross-domain generalization performance of FDACL, independent sensitivity experiments are carried out on the CWRU bearing dataset. The candidate values of λ are uniformly set as $\left\{1\times 10^{-4},5\times 10^{-4},1\times 10^{-3},5\times 10^{-3},1\times 10^{-2}\right\}$.

**Figure 13:**
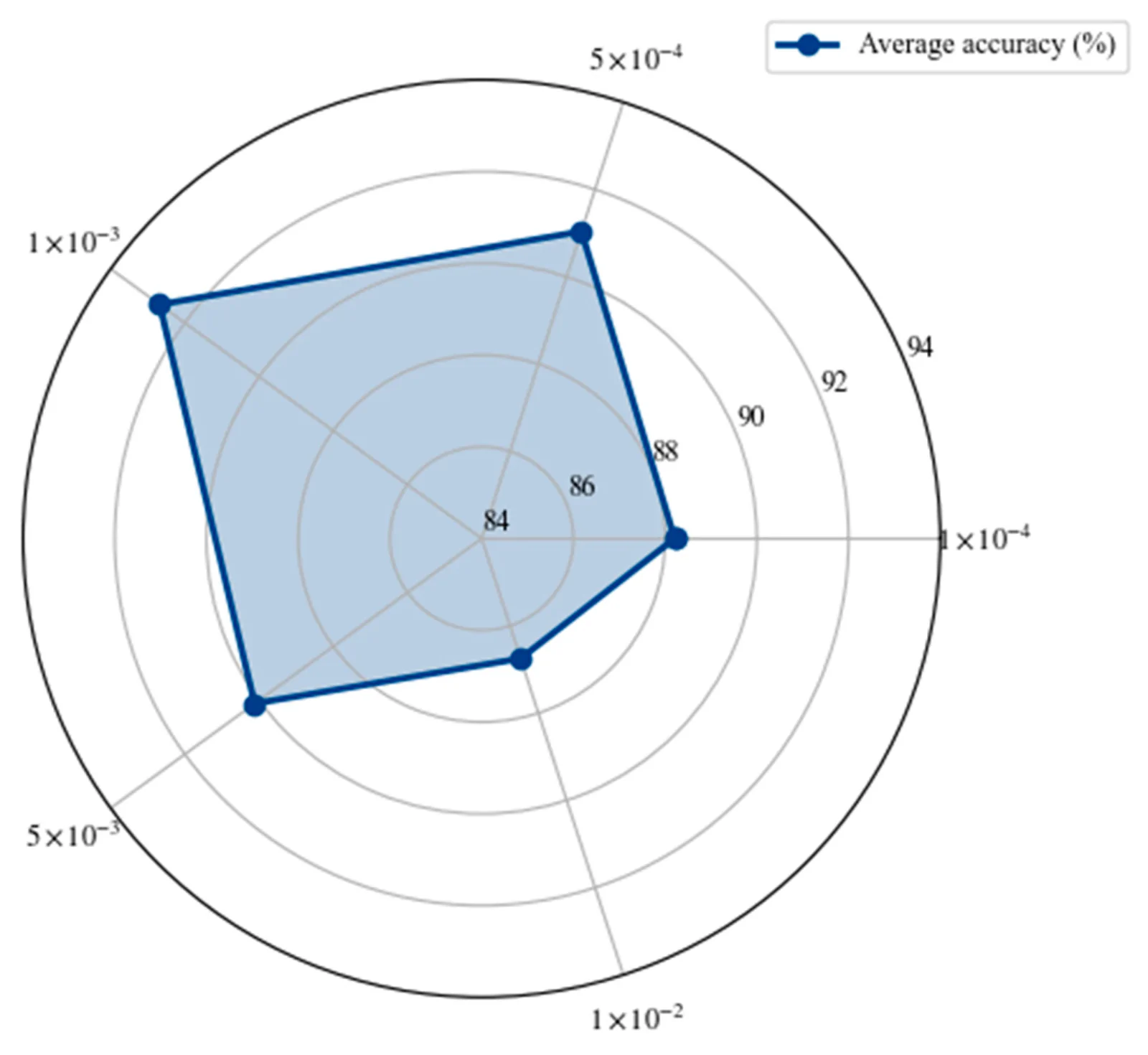
Sensitivity analysis of the balance coefficient λ on the CWRU dataset.

For the CWRU dataset, the average cross-domain diagnostic accuracy under different λ values is recorded in Figure 13. It can be observed that when λ is lower than $\lambda =1\times 10^{-3}$, insufficient regularization fails to constrain the over-concentration of component scoring weights, resulting in weak domain-invariant feature extraction and low generalization accuracy. When λ increases to $\lambda =1\times 10^{-3}$, the regularization strength reaches a reasonable balance, and FDACL achieves the highest average classification accuracy on all cross-load transfer tasks. If λ is further increased beyond $\lambda =1\times 10^{-3}$, an excessive weight penalty overly suppresses the amplitude difference in frequency importance weights, which damages the model’s ability to distinguish fault-critical spectral components and gradually reduces diagnostic performance. Within the range $5\times 10^{-4}\sim 5\times 10^{-3}$, the accuracy fluctuation is controlled within a narrow interval, which verifies that the model does not present strong sensitivity to λ on the CWRU dataset.

### 5.3. Case Study 2: Paderborn University Bearing Dataset

#### 5.3.1. Experimental Results and Analysis

The detailed partition of all single-source generalization transfer tasks on the PU dataset is summarized in Table 7. All tasks uniformly conduct classification for six distinct bearing health conditions, including the normal condition, inner race fault, outer race fault, and rolling element fault, with different damage severities (0–5).

We systematically conduct a total of 12 cross-working-condition transfer tasks based on the Paderborn University (PU) bearing dataset, which covers multiple combinations of varying rotational speeds, radial forces, and load torques. The averaged ten-run diagnostic accuracy of all comparative methods on the four groups of cross-domain transfer subtasks is summarized in Table 8. To facilitate intuitive comparison and quantitative analysis of model performance, we adopt uniform formatting rules for the table: the highest average classification accuracy on each source-target transfer task is highlighted in bold font, while the suboptimal second-best accuracy value is marked with a solid underline, making the performance ranking of all algorithms straightforward to identify at a glance.

**Figure 14:**
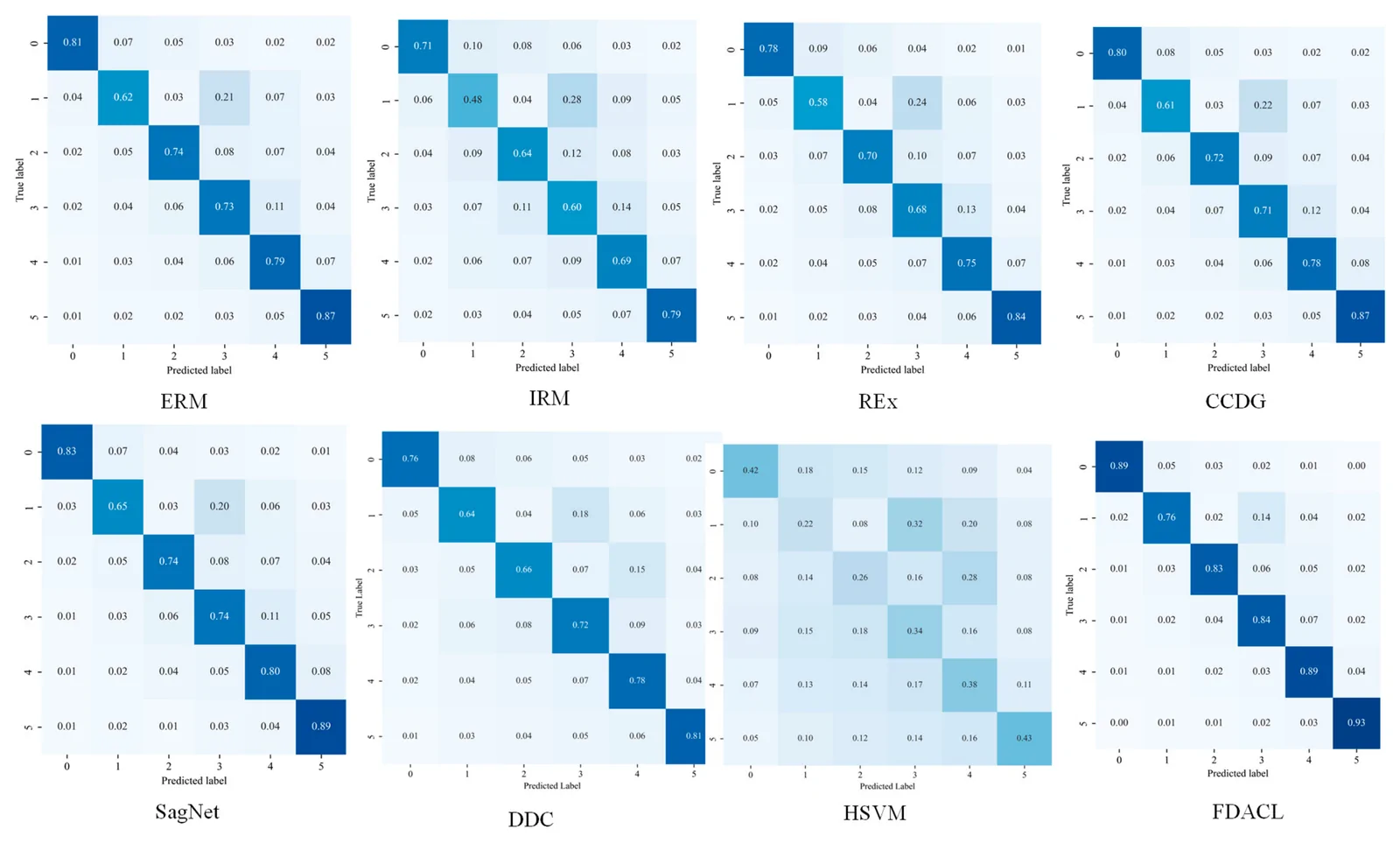
Confusion matrices for task B3 on the PU dataset.

Taking transfer task B3 as a representative example, the classification performance can be further analyzed from the perspective of the confusion matrix, as illustrated in Figure 14. Under this cross-domain transfer scenario, the proposed FDACL achieves the highest diagnostic accuracy of 84.85 ± 2.59 %, outperforming ERM, IRM, REx, CCDG and SagNet by 8.42%, 18.60%, 11.64%, 9.40% and 7.26%, respectively. As observed from the confusion matrices, conventional domain generalization methods suffer from severe inter-class confusion, especially for fault categories with similar spectral characteristics, which leads to degraded generalization performance on unseen target domains. By contrast, FDACL yields higher diagonal values and lower off-diagonal elements in the confusion matrix. It effectively suppresses misclassification among easily confused fault classes, demonstrating that the adaptive frequency-domain augmentation module helps the model capture discriminative fault-related spectral features and mitigate feature aliasing caused by domain shifts.

We further evaluate the computational overhead on the PU dataset with severe cross-domain distribution shifts, as summarized in Table 9. All deep learning methods adopt the identical encoder backbone, so they maintain equal total parameters (7.50 K) and inference FLOPs (453.03 M), consistent with the CWRU setup. For all end-to-end baselines (ERM, IRM, REx, CCDG, SagNet, DDC), the entire network undergoes gradient updates, resulting in 7.50 K trainable parameters, higher peak GPU memory usage and longer training durations. By contrast, FDACL freezes the full pre-trained backbone and only optimizes the linear classification head, cutting trainable parameters down to merely 0.65 K. The efficiency improvement in FDACL is exclusively reflected in the training backpropagation phase, while the inference computational cost stays the same as all comparative deep models. The traditional HSVM method requires no neural network computation and achieves the shortest training time (13.24 s), yet it cannot extract deep fault features and suffers from extremely poor cross-domain diagnosis accuracy. Owing to larger domain discrepancy on the PU dataset, the peak GPU memory and training time of all deep learning models rise moderately relative to the CWRU dataset. Even under this challenging setting, FDACL still yields remarkably lower peak GPU memory and shorter training time than every end-to-end domain generalization baseline. This outcome demonstrates that our two-stage training framework effectively reduces training-side computational overhead and is highly applicable to practical industrial bearing fault diagnosis.

#### 5.3.2. Hyperparameter Sensitivity Analysis

**Figure 15:**
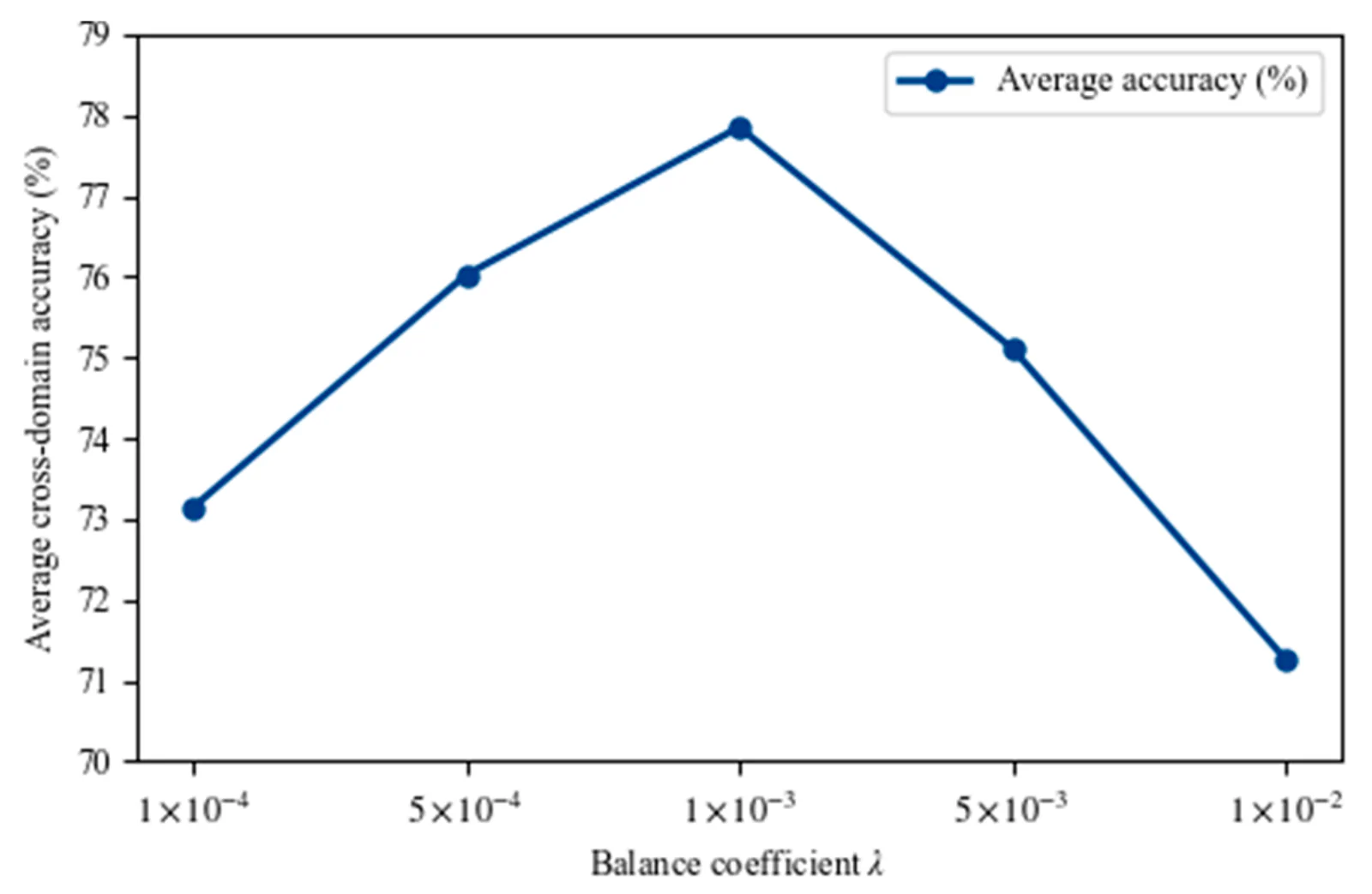
Sensitivity analysis of the balance coefficient λ on the PU dataset.

Independent sensitivity experiments are further conducted on the PU dataset with more obvious cross-domain distribution shifts, and the corresponding accuracy curve is plotted in Figure 15. Similar variation trends can be observed compared with the CWRU dataset, and the unified hyperparameter configuration consistent with CWRU is adopted for all PU experimental groups. When $\lambda =1\times 10^{-3}$, the model gains the optimal overall generalization performance across all cross-speed and cross-load transfer tasks of PU. Too small a λ value leads to overfitting on single-source vibration samples, while an overly large λ weakens the adaptive discrimination capacity of the AFA module. Compared with the CWRU dataset, the accuracy fluctuation range of the PU dataset under varying λ is slightly wider, which is attributed to the larger domain discrepancy of working conditions. Nevertheless, the model still maintains stable accuracy near the optimal λ and will not collapse due to minor parameter deviations. Combining the experimental results of two datasets, $\lambda =1\times 10^{-3}$ is selected as the unified hyperparameter configuration for all comparative and ablation experiments in this paper.

### 5.4. Case Study 3: Wheelset Bearing Dataset from CRRC Qingdao Sifang

#### 5.4.1. Experimental Results and Analysis

All comparative models adopted in this case study are consistent with the baseline methods employed in the previous case study, ensuring fair and unified experimental evaluation criteria across different datasets. The detailed task division and domain configuration of all cross-speed transfer tasks conducted on the CRRC Qingdao Sifang wheelset bearing dataset are summarized in Table 10. The quantitative DG diagnosis results of the proposed FDACL and other state-of-the-art baseline models under all transfer scenarios are illustrated in Table 11. Overall, the proposed FDACL achieves optimal or suboptimal diagnostic accuracy in the vast majority of cross-working-condition transfer tasks and yields stable and superior generalization performance compared with other competing methods. This phenomenon fully demonstrates the outstanding feature alignment ability and robust cross-domain adaptation capability of the proposed method. Specifically, FDACL exhibits distinct advantages in numerous challenging cross-speed generalization scenarios with severe data distribution shifts caused by rotational speed variations. For instance, in the transfer task where Domain 1 is treated as the source domain and Domain 0 as the unseen target domain, FDACL achieves a high diagnostic accuracy of 98.97%, which substantially exceeds the performance of the second-best SagNet method and obtains a significant performance improvement. Similarly, in the transfer task from source Domain 2 to target Domain 1, FDACL still maintains a promising accuracy of 96.54%, consistently outperforming other supervised baseline approaches. These comprehensive experimental results further validate that FDACL can effectively suppress negative interference from speed-induced domain discrepancies and extract stable, discriminative, and domain-invariant fault features for reliable cross-domain bearing fault diagnosis. Nevertheless, FDACL suffers a mild accuracy drop on tasks with huge speed gaps and heavy low-frequency noise. The current AFA module only optimizes spectral amplitude adjustment without dedicated time-domain denoising operations, so severe noise will partially obscure weak fault harmonics and cause slight feature aliasing. In contrast, all compared baselines adopt blind global transformation and fail to separate fault-sensitive frequency bands, leading to much more drastic performance decline under large distribution shifts.

Similarly, for the transfer task of generalizing from source Domain 2 to target Domain 1, FDACL reaches an accuracy of 96.54%, significantly higher than all competing baselines. These experimental results demonstrate that FDACL can effectively mitigate distribution discrepancies induced by rotational speed variations and enable robust extraction of discriminative fault features.

**Figure 16:**
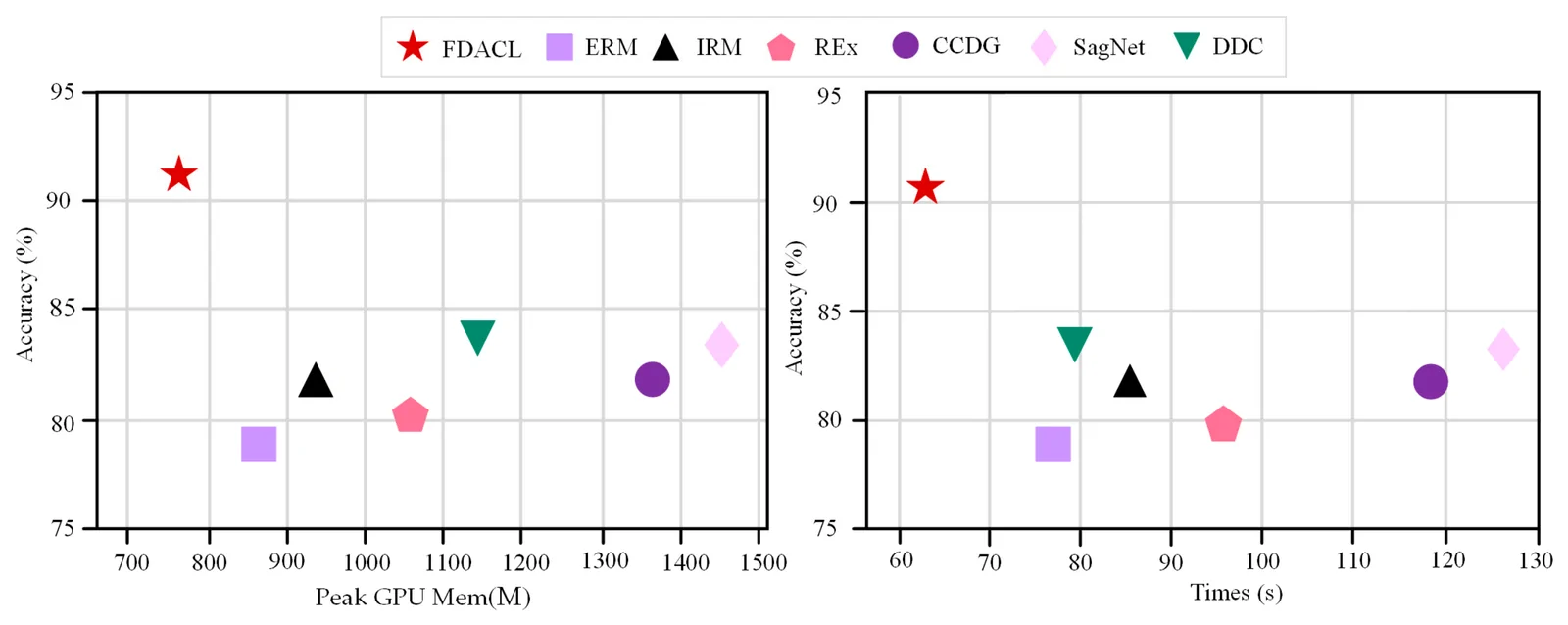
Computational complexity of the model on the Qingdao Sifang bearing dataset.

Figure 16 presents the Pareto-front comparison between diagnostic accuracy and computational overhead for all competing approaches. The left panel illustrates the trade-off between classification accuracy and peak GPU memory consumption, while the right panel describes the relationship between accuracy and total training time. It can be observed that most comparative methods occupy the right-hand region of the coordinate plane, which indicates that they require higher GPU memory resources or longer training duration to achieve competitive diagnostic results. By contrast, the red star marker corresponding to FDACL is located in the upper-left area of both panels. This position signifies that FDACL yields the highest diagnostic accuracy, meanwhile maintaining low peak GPU memory and short training time. Benefiting from the two-stage training scheme with a frozen backbone encoder, only the lightweight linear classifier head is updated during downstream adaptation, effectively reducing the volume of parameters participating in gradient backpropagation. The above results demonstrate that FDACL achieves an optimal Pareto balance between diagnostic performance and training-side computational cost, showing great application potential for practical bearing fault diagnosis scenarios.

**Figure 17:**
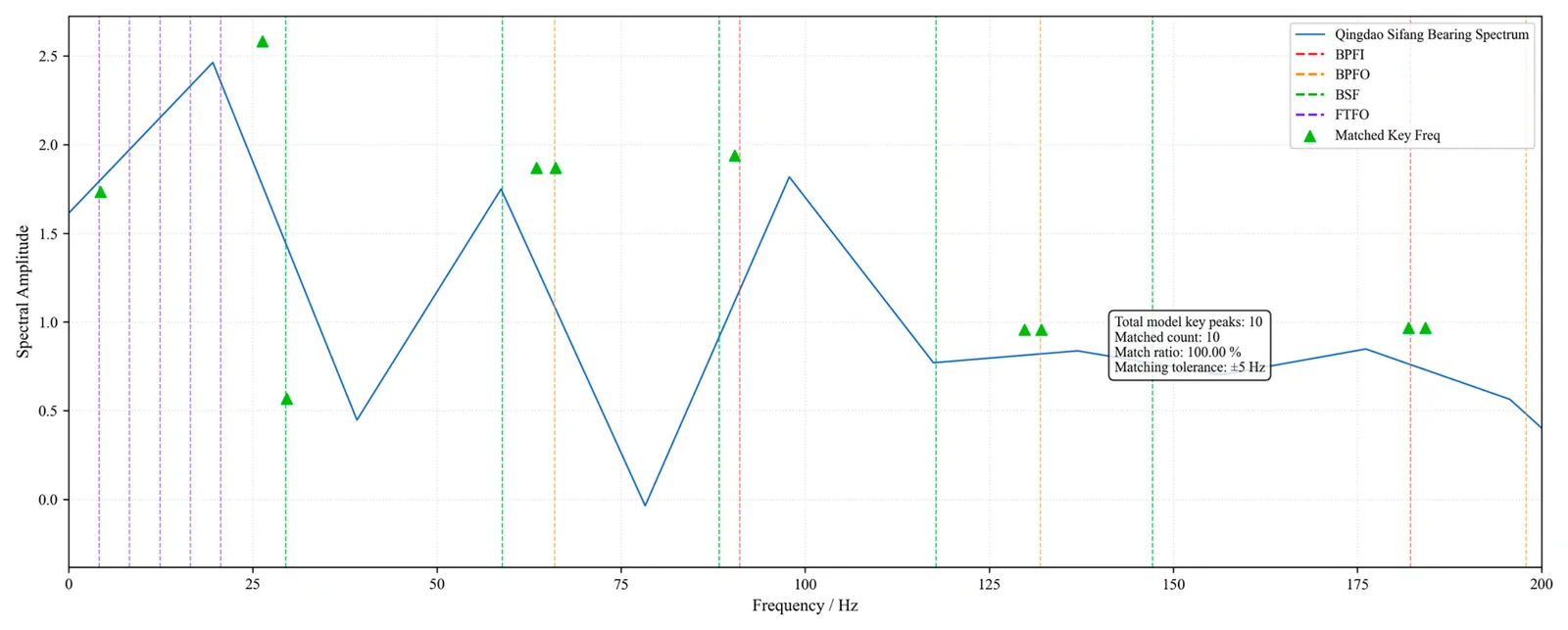
Matching verification between AFA-extracted key frequencies and theoretical bearing fault frequencies.

To verify the physical interpretability of the learnable frequency weight vector in the AFA module, we quantitatively match the top-10 high-attention frequency peaks extracted by the model against the theoretical geometric fault frequencies of the CRRC Qingdao Sifang wheelset bearing. Based on the bearing geometric parameters (d=26mm, D=160mm, Z=16, $\alpha =10^{{}^{\circ}}$) and the operating speed (n=589 r/min), four theoretical characteristic frequencies and their 1–5-order harmonics are calculated: inner race fault frequency BPFI = 91.10 Hz, outer race fault frequency BPFO = 65.97 Hz, rolling element fault frequency BSF = 29.43 Hz, and cage fault frequency FTFO = 4.12 Hz. A frequency tolerance window of ±5 Hz is defined as the matching judgment threshold. The spectrum-matching verification in Figure 17 is shown below, where dashed lines with different colors correspond to the theoretical fault characteristic frequencies of four failure modes, and green triangular markers denote the key frequency peaks extracted by the AFA module. As counted in the text box on the graph, all 10 high-attention dominant frequency components extracted by the model are successfully matched to the theoretical fault fundamental frequencies or their integer harmonics within the tolerance range, achieving a 100% matching rate. This perfect one-to-one matching relationship fully proves that the adaptive frequency enhancement (AFA) module possesses strong physical interpretability. It can autonomously screen out frequency components corresponding to real bearing fault signatures from the full-band spectrum, instead of capturing meaningless random noise. This conclusion provides reliable physical mechanism support for the proposed FDACL model in the field of railway axle box bearing intelligent fault diagnosis.

**Figure 18:**
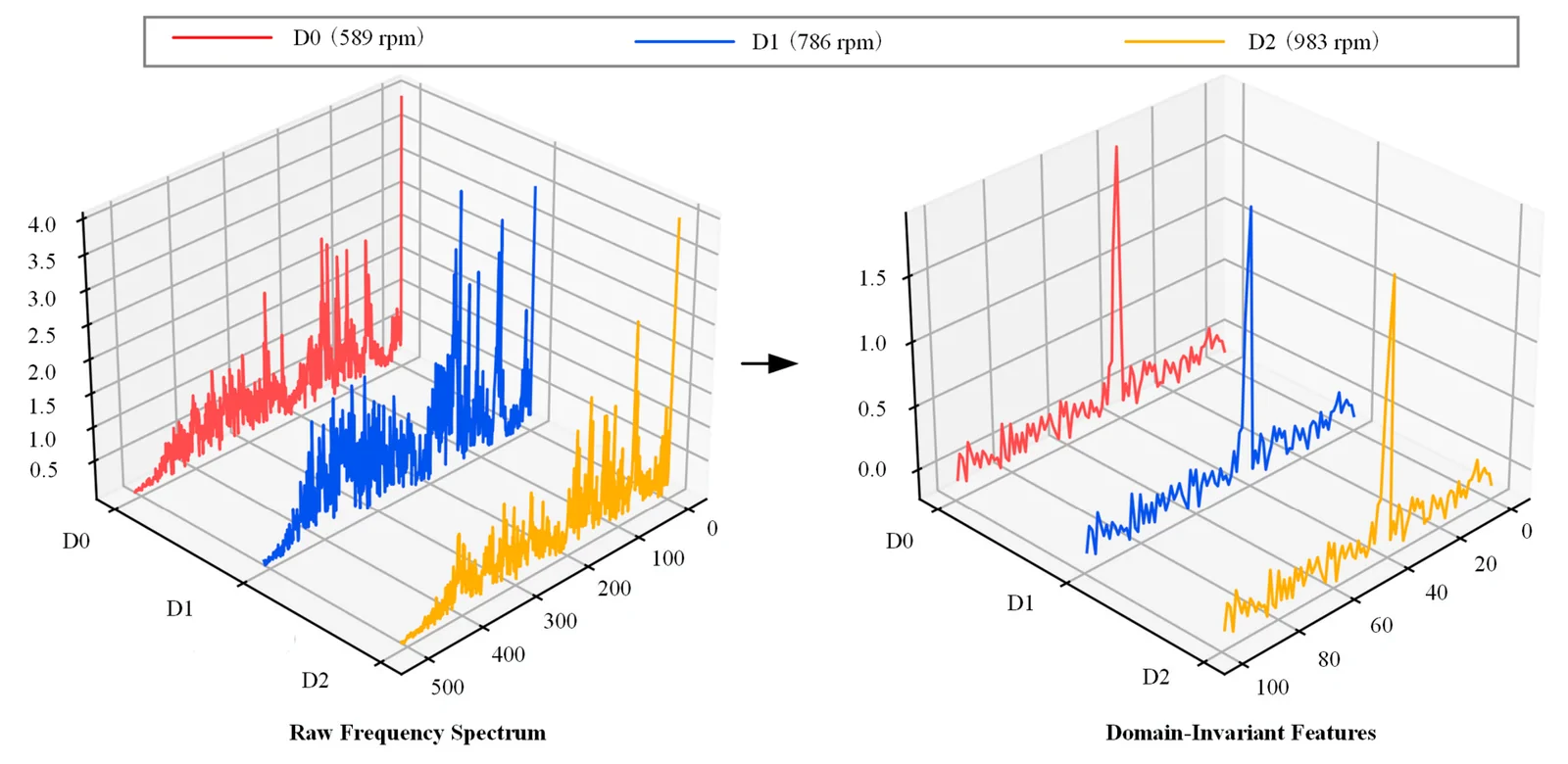
Feature-encoding results of the proposed FDACL method.

To deeply interpret the DG performance of the proposed FDACL, this paper conducts a visualization analysis on raw samples and their feature vectors encoded by the model. Figure 18 presents frequency-domain signal samples under distinct rotational speeds and the corresponding feature vectors encoded by FDACL for identical fault types based on the wheelset bearing dataset from CRRC Qingdao Sifang. In the left three-dimensional plot, the red, blue, and yellow curves denote samples from the source domain D0 and two target domains D1 and D2, respectively. It can be intuitively observed that obvious discrepancies in amplitude and waveform exist among raw signals collected under different rotating speeds. In contrast, after encoding via FDACL, the corresponding feature vectors overlap heavily and are nearly identical in the feature space. This visualization result demonstrates that FDACL can effectively align feature representations of samples originating from distinct working-condition domains, which further reveals the internal mechanism behind the outstanding performance of the model on various DG tasks.

#### 5.4.2. Ablation Experiment

Systematic ablation experiments are conducted in this study to validate the contribution of each core component within the proposed FDACL framework. Four classic frequency-domain data augmentation schemes are chosen as comparative baselines, abbreviated as FN, FM, FW, and FR, respectively. Specifically, FN injects Gaussian white noise with an SNR range of 10–20 dB into frequency-domain signals; FM randomly masks 20–80% spectral components; FW performs global amplitude scaling on the full spectrum with a scaling factor ξ∈(0,5); and FR applies independent random amplitude scaling r∈(0,5) to each frequency component. All four baseline methods boost data diversity via blind, unweighted amplitude distortion over the whole frequency spectrum.

As summarized in Table 12, extra ablation variants are further constructed to quantitatively analyze the independent contribution of each core component. The four frequency augmentation baselines (FN, FM, FW, FR) replace the proposed AFA module one by one, which directly reveals the performance superiority brought by the adaptive frequency band screening mechanism of AFA. The AE variant removes the unsupervised InfoNCE contrastive loss and only adopts supervised ERM loss for training, so the accuracy gap between AE and full FDACL fully demonstrates the critical generalization promotion of contrastive learning. In addition, the AT variant transfers the AFA module to raw time-domain vibration signals without frequency conversion. The obvious accuracy decline in AT proves that the designed AFA module is specially tailored for frequency-domain vibration features and possesses strong frequency-targeted optimization capability.

To verify the necessity of unsupervised contrastive learning for SDG, we construct the ERM and AE ablation variants. The ERM variant removes the entire AFA module and only adopts supervised empirical risk minimization training. The AE variant retains AFA while disabling contrastive learning, where model training relies solely on ERM loss after AFA. Furthermore, the AT variant is designed to investigate the generalization of the AFA module, which directly applies the AFA operation to raw time-domain vibration signals rather than frequency-domain spectra. Table 12 provides complete definitions for all baseline and ablation configurations, covering FN, FM, FW, FR, ERM, AE, and AT.

The proposed AFA module appears structurally similar to the combination of FW and FR, but its core mechanism is essentially distinct from conventional augmentation operations. Instead of applying identical random perturbations to all frequency bins, AFA learns trainable weights to evaluate the discriminative importance of each frequency component, splitting the spectrum into fault-critical frequency bands and trivial non-critical bands. Mild random perturbations are imposed on critical frequencies to retain intrinsic fault semantic information, whereas heavy random disturbances are assigned to non-critical bands to mimic distribution shifts induced by varying operating speeds. As a result, AFA acts as an adaptive generative augmentation strategy that balances fault semantic preservation and cross-domain distribution expansion, instead of a simple unweighted spectral transformation.

**Figure 19:**
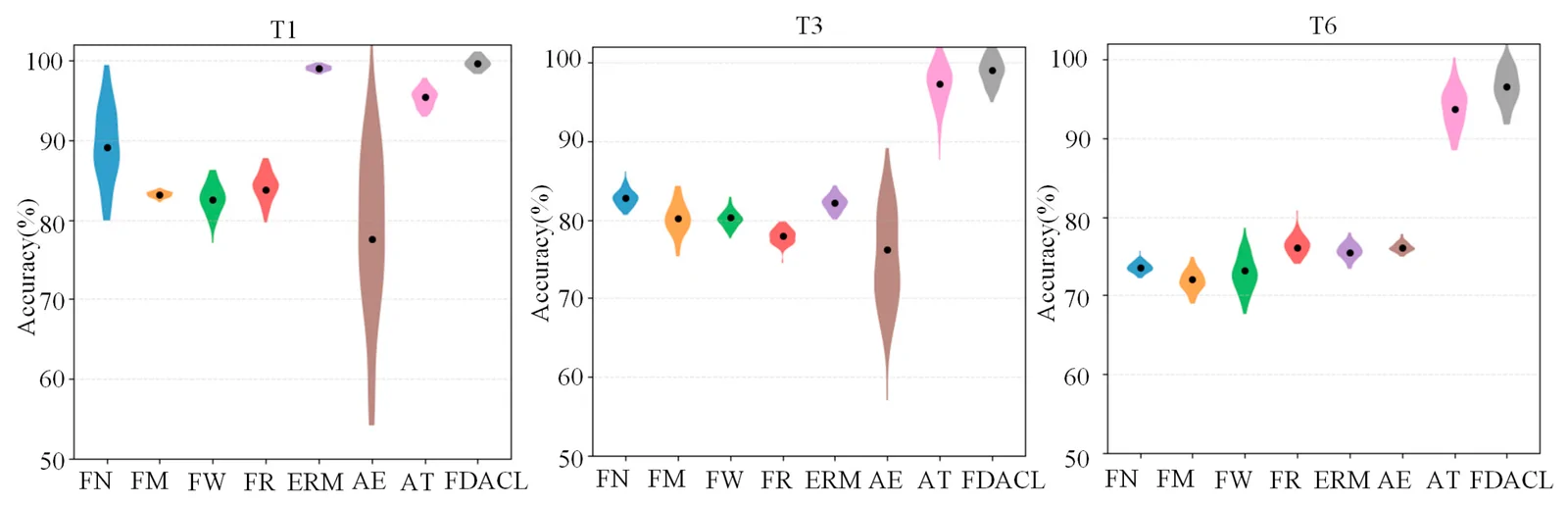
Results of the ablation studies.

The quantitative distribution results of diagnostic accuracy are illustrated in Figure 19. The proposed FDACL achieves state-of-the-art performance across all three cross-working-condition transfer tasks (T1, T3 and T6), with its accuracy distribution tightly concentrated at high values and minimal dispersion, which strongly demonstrates the outstanding cross-domain generalization capacity of the proposed framework. The violin plots of ablation variants explicitly illustrate the sources of FDACL’s superior performance from three aspects:

First, comparing the AT variant with FDACL, we observe that the AFA module customized for frequency-domain signals delivers far better performance than directly applying augmentation operations to raw time-domain vibration signals. For bearing vibration signals, fault-related information is embedded in a set of characteristic harmonics and sidebands in the frequency domain, where the relative amplitude relationships among these spectral components carry critical fault physical semantics. When the amplitude-adjusting AFA operation is directly imposed on raw time-domain waveforms, the temporal perturbation cannot selectively act on individual frequency components. Such time-domain random scaling will arbitrarily disrupt the inherent amplitude ratios among fault characteristic frequencies, sidebands, and rotational harmonics, thereby destroying the physical correlation of fault features and contaminating fault semantic information. In contrast, performing amplitude perturbation in the frequency domain supports band-wise independent manipulation. We can apply mild perturbation to fault-dominant spectral lines to retain their relative amplitude relationships, while introducing large-range disturbance to noise-dominated irrelevant frequency bands. This frequency-selective manipulation preserves the physical harmonic structure of bearing faults, which accounts for the substantially better generalization of frequency-domain AFA over its time-domain AT counterpart.

Second, significant accuracy degradation can be observed in ERM and AE after removing unsupervised contrastive learning. The overall accuracy distribution shifts downward with distinctly reduced mean values, which confirms that unsupervised contrastive learning serves as the core constraint for learning domain-invariant discriminative fault features.

Third, compared with four conventional frequency-domain random augmentation baselines (FN, FM, FW, FR) that impose blind uniform spectral disturbances and only yield marginal performance gains, the combination of AFA and unsupervised contrastive learning enables the model to automatically distinguish fault-critical frequency components from trivial, irrelevant bands. This joint design effectively mitigates severe distribution discrepancies induced by varying operating speeds, and consequently yields the best diagnostic performance on all cross-domain transfer tasks.

## 6. Comparative Analysis with Additional State-of-the-Art Papers

Considering that the CWRU bearing dataset used in this paper has been widely applied in existing cross-domain fault diagnosis research, we selected five representative, recently published studies focusing on variable-load bearing fault diagnosis based on the CWRU dataset for comprehensive horizontal comparison, including TAD-Mix [35], AG-WPEA-DC [36], PKECA [37], DEMDGN [38], and DFS-DG [39]. All cross-domain transfer experimental settings are unified to be consistent with our work, and the average diagnostic accuracy reported in each study is summarized in Table 13.

As illustrated in Table 13, the average diagnostic accuracy of the proposed FDACL reaches 92.68% across all cross-load transfer tasks on the CWRU bearing dataset, showing competitive overall performance compared with five mainstream SOTA variable-load fault diagnosis methods. PKECA and TAD-Mix achieve higher average accuracies of 95.38% and 94.72%, relying on global distribution matching and intensive time-domain data augmentation to mitigate overall domain discrepancies. Differently, FDACL integrates adaptive frequency enhancement and contrast learning to capture subtle fault frequency information from raw vibration signals. Even though its overall average accuracy is marginally inferior to PKECA and TAD-Mix, FDACL achieves optimal results on transfer tasks with distinct fault frequency discrepancies and outperforms DEMDGN and DFS-DG in average accuracy.

## 7. Conclusions

This chapter summarizes the core work, key experimental findings and engineering value of the proposed Frequency-Domain Adaptive Contrastive Learning (FDACL) framework. Consistent conclusions derived from three distinct bearing datasets are summarized, the practical advantages under scarce labeled data are discussed, and existing research limitations and future research directions are outlined.

### 7.1. Global Discussion

Across the CWRU, PU and CRRC wheelset bearing datasets, FDACL exhibits consistent advantages. Unlike supervised domain generalization models that rely on a large amount of labeled data, FDACL implements fully unsupervised pre-training and only requires unlabeled single-source vibration signals, which significantly reduces the data labeling cost in industrial scenarios. The specially designed AFA module can distinguish fault-critical frequency components from noise-dominated bands and implement targeted perturbation rather than blind full-spectrum disturbance, thus preserving inherent fault harmonic information during sample augmentation. Moreover, the two-stage training strategy drastically reduces the number of trainable parameters in the fine-tuning phase, cutting GPU memory usage and training duration while sustaining stable cross-domain generalization capacity, making the method more compatible with embedded industrial diagnostic devices. In addition, FDACL only suffers mild accuracy degradation under extreme small-sample settings, whereas most comparative models experience sharp performance drops, which demonstrates its strong adaptability to sparse data. Nevertheless, comprehensive experimental results on multiple datasets also reveal several obvious shortcomings of the proposed model. The frequency discrimination logic of the AFA module is only developed for single-channel, one-dimensional vibration signals, without corresponding processing strategies for multi-channel mixed monitoring signals. In addition, the hyperparameters controlling frequency perturbation intensity need manual tuning for different sampling frequencies, and the model lacks a fully self-adaptive optimization mechanism. Furthermore, the feature alignment capability of FDACL declines remarkably when there exists an enormous mechanical structure gap between source and target domains, restricting its applicability across entirely distinct types of mechanical equipment.

Despite the competitive generalization performance achieved by the proposed FDACL across three bearing datasets, this work still has an inherent physical limitation in the design of the AFA module, which we explicitly discuss here for objectivity. The current AFA module only adopts differentiated amplitude perturbation to simulate the cross-domain discrepancies of vibration signals, while it lacks independent frequency-axis scaling operations to replicate the core physical law that rotational speed variation shifts bearing fault characteristic frequencies. In real rotating machinery systems, different speeds directly change the fundamental frequency and harmonic positions of fault signatures; simple amplitude adjustment cannot fully reproduce such frequency migration phenomena. Therefore, when the target domain has an extremely large speed gap relative to the source domain and obvious frequency offset exists, the data samples generated by AFA cannot completely fit the real spectral distribution of unseen working conditions, leading to the slight diagnostic accuracy decline observed in several difficult cross-speed transfer tasks. In future research, we will integrate frequency stretching/compression transformation into the augmentation pipeline to jointly simulate both amplitude and frequency shifts caused by variable speeds, further improving the physical completeness of the augmentation strategy. Apart from this core limitation, the AFA module is only applicable to single-channel vibration signals at present and requires manual adjustment of spectrum segmentation thresholds for different sampling frequencies, which will also be optimized in follow-up studies.

### 7.2. Concluding Remarks

This subsection concludes the core contributions, experimental results and engineering significance of FDACL. It summarizes unified laws from three bearing datasets and proposes future improvements against the model’s drawbacks.

Aiming to address label scarcity and accuracy drops under varying working conditions, this work builds the FDACL single-source generalization model by combining adaptive frequency augmentation and unsupervised contrastive learning. The AFA module distinguishes fault-related frequency bands with learnable weights and generates diverse pseudo samples via differentiated perturbation. Guided by InfoNCE loss, the model extracts speed- and load-robust fault features without any target-domain labels.

Validated on the CWRU, PU and CRRC datasets, FDACL matches or outperforms mainstream supervised DG methods on most cross-working tasks and maintains stable performance under extremely limited samples. Visualization and ablation tests confirm the necessity of each component. This method provides a low-label, high-generalization solution for industrial bearing diagnosis.

## Figures and Tables

**Table 1:** Parameters of the CWRU bearing dataset.

Health Condition	Domain Label	Load (HP)	Rotational Speed (rpm)	Sampling Frequency (kHz)	Fault Severity (mm)
NA, IR, OR, RF	0	0	1797	12 kHz	0.1778
	1	1	1772		0.3556
	2	2	1750		0.5334
	3	3	1730		0.1778

**Table 2:** Operating condition configurations of the PU dataset.

Domain Label	Load Torque (Hm)	Radial Force (N)	Speed (rpm)
0	0.7	1000	1500
1	0.7	1000	900
2	0.1	1000	1500
3	0.7	400	1500

**Table 3:** Parameters of Qingdao Sifang bearing dataset.

Health Condition	Domain Label	Speed (rpm)	Sampling Frequency (kHz)	Fault Severity (mm)
NA, IR, OR, RF	0	589	20	IR: 10 × 30 OR: 10 × 45 RF: 3 × 35
	1	786		
	2	983		

**Table 4:** Transfer task details of the CWRU bearing dataset.

Task Number	Source Domain	Target Domain	Label
A1	0	1	C0~C9
A2	0	2	
A3	0	3	
A4	1	0	
A5	1	2	
A6	1	3	
A7	2	0	
A8	2	1	
A9	2	3	
A10	3	0	
A11	3	1	
A12	3	2	

**Table 5:** Diagnostic results of the CWRU dataset. (Note: Values in bold represent the optimal results, and underlined values represent the suboptimal results.)

Task	Method							
	ERM (%)	IRM (%)	REx (%)	CCDG (%)	SagNet (%)	DDC (%)	HSVM (%)	FDACL (%)
A1	**98.41 ± 0.40**	98.14 ± 0.77	97.91 ± 1.51	96.01 ± 1.05	96.78 ± 0.99	95.64 ± 1.02	88.21 ± 2.14	97.65 ± 1.08
A2	93.84 ± 1.48	93.11 ± 2.78	91.73 ± 5.80	88.29 ± 2.99	91.96 ± 1.93	**96.07 ± 0.09**	85.14 ± 2.21	93.27 ± 1.74
A3	68.22 ± 3.14	69.41 ± 4.17	67.16 ± 8.66	64.71 ± 5.83	67.32 ± 4.48	**89.96 ± 2.01**	68.47 ± 2.14	75.56 ± 5.01
A4	**96.98 ± 1.25**	95.95 ± 0.54	96.36 ± 0.78	94.82 ± 2.12	94.12 ± 1.94	92.68 ± 4.02	85.41 ± 0.07	93.66 ± 2.64
A5	99.24 ± 0.46	98.72 ± 1.62	99.23 ± 1.09	98.44 ± 1.19	**99.47 ± 0.46**	98.01 ± 1.21	84.01 ± 3.25	97.90 ± 1.05
A6	84.54 ± 3.68	83.17 ± 3.59	85.82 ± 4.06	85.60 ± 4.26	87.51 ± 3.25	96.45 ± 3.14	77.65 ± 5.21	**89.26 ± 3.44**
A7	94.03 ± 1.12	93.95 ± 1.22	93.24 ± 2.12	92.44 ± 1.54	91.02 ± 1.49	94.01 ± 2.21	79.64 ± 1.04	**95.05 ± 1.37**
A8	**98.36 ± 0.15**	97.78 ± 0.92	98.11 ± 0.45	97.07 ± 0.79	98.23 ± 0.38	96.69 ± 1.24	93.07 ± 6.07	97.05 ± 0.73
A9	97.39 ± 0.70	97.12 ± 0.76	96.42 ± 3.87	91.43 ± 2.90	97.78 ± 0.62	96.54 ± 4.48	89.25 ± 8.50	**98.45 ± 0.96**
A10	84.75 ± 3.83	84.30 ± 2.91	83.16 ± 3.45	84.63 ± 2.69	82.70 ± 3.10	84.65 ± 2.01	67.25 ± 3.47	**86.38 ± 1.83**
A11	**93.71 ± 1.39**	91.99 ± 1.91	93.19 ± 1.70	92.77 ± 1.13	93.51 ± 1.95	92.01 ± 3.47	90.21 ± 2.57	91.39 ± 1.99
A12	94.39 ± 1.31	93.45 ± 1.49	93.19 ± 1.28	92.31 ± 0.84	93.65 ± 1.48	93.03 ± 0.34	88.75 ± 0.26	**96.57 ± 1.64**

**Table 6:** Comparison of computational overhead among different domain generalization methods in the CWRU dataset. (Note: Values in bold represent the optimal results.)

Model	Total Params (K)	Trainable Params (K)	FLOPs (M)	Peak GPU Mem (MB)	Time (s)
ERM	7.50	7.50	453.03	1426	82.29
IRM	7.50	7.50	453.03	1542	157.88
REx	7.50	7.50	453.03	1564	161.45
CCDG	7.50	7.50	453.03	1580	163.71
SagNet	7.50	7.50	453.03	1572	164.24
DDC	7.50	7.50	453.03	1530	155.32
HSVM	-	-	-	-	12.35
FDACL	7.50	**0.65**	453.03	**812**	**69.27**

**Table 7:** Transfer task details of the PU bearing dataset.

Task Number	Source Domain	Target Domain	Label
B1	0	1	0~5
B2	0	2	
B3	0	3	
B4	1	0	
B5	1	2	
B6	1	3	
B7	2	0	
B8	2	1	
B9	2	3	
B10	3	0	
B11	3	1	
B12	3	2	

**Table 8:** Diagnostic results of the PU dataset. (Note: Values in bold represent the optimal results, and underlined values represent the suboptimal results.)

Task	Method							
	ERM (%)	IRM (%)	REx (%)	CCDG (%)	SagNet (%)	DDC (%)	HSVM (%)	FDACL (%)
B1	21.79 ± 2.57	28.34 ± 6.26	28.55 ± 2.39	32.66 ± 1.44	32.16 ± 2.50	30.52 ± 3.12	41.26 ± 4.35	**69.30 ± 4.69**
B2	90.42 ± 2.05	82.73 ± 5.85	88.85 ± 2.97	**91.16 ± 1.31**	90.74 ± 0.64	85.37 ± 4.21	48.71 ± 3.92	86.05 ± 3.16
B3	76.43 ± 2.45	67.25 ± 8.34	73.21 ± 3.87	75.45 ± 2.68	77.59 ± 1.73	78.62 ± 3.58	46.33 ± 5.17	**84.85 ± 2.59**
B4	42.29 ± 5.19	35.51 ± 9.68	40.85 ± 4.95	39.45 ± 3.90	50.33 ± 5.03	48.76 ± 4.02	40.15 ± 3.64	**70.67 ± 6.07**
B5	50.55 ± 4.03	39.35 ± 9.01	46.76 ± 3.73	47.93 ± 4.12	54.60 ± 2.46	52.18 ± 3.85	43.89 ± 4.08	**74.51 ± 2.74**
B6	42.17 ± 3.33	32.34 ± 7.18	39.51 ± 2.94	41.59 ± 3.15	46.31 ± 3.68	44.27 ± 3.69	42.07 ± 3.31	**71.20 ± 3.91**
B7	93.93 ± 1.41	71.19 ± 1.66	93.27 ± 0.65	93.78 ± 1.41	**94.58 ± 1.47**	92.35 ± 2.17	49.24 ± 2.86	91.01 ± 1.49
B8	33.19 ± 2.18	31.74 ± 6.70	35.95 ± 2.91	35.12 ± 1.70	37.64 ± 2.08	36.82 ± 3.25	40.68 ± 4.73	**65.97 ± 5.54**
B9	79.18 ± 2.17	59.64 ± 1.61	77.82 ± 3.37	77.46 ± 2.72	77.34 ± 2.09	78.56 ± 2.94	45.12 ± 3.45	**82.38 ± 2.38**
B10	80.99 ± 0.87	64.04 ± 1.24	81.33 ± 0.91	81.67 ± 1.20	82.70 ± 1.39	82.15 ± 2.68	47.56 ± 3.02	**87.72 ± 1.83**
B11	39.57 ± 1.86	38.02 ± 4.88	42.24 ± 2.71	40.54 ± 1.27	42.89 ± 2.26	41.36 ± 3.07	41.93 ± 4.29	**70.05 ± 5.20**
B12	75.28 ± 2.03	68.17 ± 6.04	75.21 ± 1.87	76.44 ± 2.07	78.42 ± 2.33	77.19 ± 2.85	44.75 ± 3.77	**80.53 ± 2.21**

**Table 9:** Comparison of computational overhead among different domain generalization methods in the PU dataset. (Note: Values in bold represent the optimal results.)

Model	Total Params (K)	Trainable Params (K)	FLOPs (M)	Peak GPU Mem (MB)	Time (s)
ERM	7.50	7.50	453.03	1426	82.29
IRM	7.50	7.50	453.03	1542	157.88
REx	7.50	7.50	453.03	1564	161.45
CCDG	7.50	7.50	453.03	1580	163.71
SagNet	7.50	7.50	453.03	1572	164.24
DDC	7.50	7.50	453.03	1545	168.2
HSVM	-	-	-	-	13.24
FDACL	7.50	**0.65**	453.03	**812**	**69.27**

**Table 10:** Transfer task details of the Qingdao Sifang bearing dataset.

Task Name	Source Domain	Target Domain	Label
T1	0	1	L0~L3
T2	0	2	
T3	1	0	
T4	1	2	
T5	2	0	
T6	2	1	

**Table 11:** Diagnostic results on the Qingdao Sifang bearing dataset. (Note: Values in bold represent the optimal results, and underlined values represent the suboptimal results.)

Task	Method							
	ERM (%)	IRM (%)	REx (%)	CCDG (%)	SagNet (%)	DDC (%)	HSVM (%)	FDACL (%)
T1	99.44 ± 0.38	78.92 ± 4.76	87.42 ± 7.94	85.81 ± 6.22	89.58 ± 6.77	90.16 ± 5.83	48.35 ± 3.21	**99.69 ± 0.80**
T2	**97.99 ± 0.88**	73.10 ± 1.32	77.68 ± 4.09	75.71 ± 3.19	77.03 ± 2.98	78.42 ± 3.56	46.72 ± 2.94	89.23 ± 9.05
T3	71.50 ± 3.31	70.00 ± 5.97	73.00 ± 1.64	69.68 ± 0.71	75.00 ± 8.63	74.27 ± 4.19	42.18 ± 4.05	**98.97 ± 1.73**
T4	75.05 ± 0.02	73.02 ± 1.51	74.64 ± 0.50	74.00 ± 1.36	75.14 ± 1.91	75.83 ± 2.64	43.64 ± 3.17	**94.47 ± 4.36**
T5	59.97 ± 1.15	65.90 ± 0.68	66.24 ± 0.27	64.41 ± 1.53	61.54 ± 0.86	63.75 ± 3.02	40.93 ± 3.66	**87.76 ± 5.17**
T6	67.82 ± 1.46	62.45 ± 1.06	62.97 ± 1.24	61.95 ± 1.55	62.36 ± 0.79	65.11 ± 2.87	41.57 ± 2.82	**96.54 ± 2.51**

**Table 12:** Descriptions of ablation method configurations.

Method	Description
FN	Randomly inject Gaussian white noise with signal-to-noise ratio ranging from 10 dB to 20 dB into frequency-domain signals
FM	Randomly mask 20% to 80% of the components of frequency-domain signals
FW	Globally scale the entire frequency-domain signal by a factor ξ, where ξ∈(0,5)
FR	Multiply each component of the frequency-domain signal by a random scalar r, r∈(0,5)
ERM	Supervised training implemented via empirical risk minimization (ERM) without the AFA module
AE	Disable unsupervised contrastive learning. Perform AFA first, followed by supervised training with ERM loss
AT	Apply the proposed AFA module to raw time-domain vibration signals

**Table 13:** Average classification accuracies of FDACL and five referenced SOTA methods on CWRU. (Note: Values in bold represent the optimal results)

Model	Brief Core Idea	Avg. Accuracy
TAD-Mix	Time-domain data augmentation	94.72
AGWPEA-DC	Fixed wavelet attention network	93.15
PKECA	Static time-frequency prior learning	**95.38**
DEMDGN	Multi-source distribution alignment	91.66
DFS-DG	Static invariant feature separation	92.41
FDACL	Adaptive frequency + contrast learning	92.68

## Data Availability

Dataset available on request from the authors. Informed consent was obtained from all subjects involved in the study.
